# Three‐dimensional distribution of wall shear stress and its gradient in red cell‐resolved computational modeling of blood flow in in vivo‐like microvascular networks

**DOI:** 10.14814/phy2.14067

**Published:** 2019-05-06

**Authors:** Peter Balogh, Prosenjit Bagchi

**Affiliations:** ^1^ Mechanical and Aerospace Engineering Department Rutgers, The State University of New Jersey Piscataway New Jersey

**Keywords:** Computational modeling, microcirculation, microvascular networks, red blood cells, wall shear stress

## Abstract

Using a high‐fidelity, 3D computational model of blood flow in microvascular networks, we provide the full 3D distribution of wall shear stress (WSS), and its gradient (WSSG), and quantify the influence of red blood cells (RBCs) on WSS and WSSG. The deformation and flow dynamics of the individual RBCs are accurately resolved in the model, while physiologically realistic microvascular networks comprised of multiple bifurcations, convergences, and tortuous vessels are considered. A strong heterogeneity in WSS and WSSG is predicted across the networks, with the highest WSS occurring in precapillary bifurcations and capillary vessels. 3D variations of WSS and WSSG are shown to occur due to both network morphology and the influence of RBCs. The RBCs increase the WSS by as much as three times compared to that when no RBCs are present, and the highest increase is observed in venules. WSSG also increases significantly, and high WSSGs occur over wider regions in the presence of RBCs. In most vessels, the circumferential component of WSSG is observed to be greater than the axial component in the presence of RBCs, while the opposite trend is observed when RBCs are not considered. These results underscore the important role of RBCs on WSS and WSSG that cannot be predicted by widely used 1D models of network blood flow. Furthermore, the subendothelium‐scale variations of WSS and WSSG predicted by the present model have implications in terms of endothelial cell functions in the microvasculature.

## Introduction

The shear stress exerted by blood on the vascular walls, often termed as the wall shear stress (WSS), is responsible for many physiological and pathological processes. A primary role of WSS in the microcirculation is the regulation of blood flow. Endothelial cells (ECs) lining the vessel walls can sense WSS (Davies 1995; Chien 2007) and trigger vessel dilation or contraction (Langille and O'Donnell 1986; dela Paz and D'Amore 2009). In the microcirculation, WSS‐mediated flow regulation primarily occurs in arterioles which are surrounded by smooth muscle cells (SMC) that respond to vasodilators and constrictors (Lu and Kassab 2011; Fleming et al. 2017). WSS also stimulates ECs to express a diverse range of ion channels, growth factors and inhibitors, adhesion molecules, and chemoattractants (Cabrales et al. 2006; Davies 2009). WSS can initiate mechanotransduction at subcellular scales by affecting the EC glycocalyx, membrane fluidity, cytoskeleton reorganization, and focal adhesion (Wang et al. 1993; Haidekker et al. 2000; Butler et al. 2001; Li et al. 2002; Thi et al. 2004; Yamamoto and Ando 2013). WSS also affects EC gap junction and permeability, thereby affecting trans‐endothelium transport as well as vascular development (Tarbell 2010). EC oxygen consumption, proliferation, migration, and apoptosis are affected by WSS (Li et al. 2005; Cabrales et al. 2006; Kadohama et al. 2007; Chien 2008; Metaxa et al. 2008). EC response to WSS is a primary driver behind angiogenesis and vascular remodeling (Gibbons and Dzau 1994; Zogakis and Libutti 2001; Carmeliet 2005).

One of the earliest theoretical works concerned with WSS is that of Murray (1926). Though originally developed to understand the design principles of vascular networks, Murray's law predicts a constant WSS throughout the vasculature. While this generally occurs in the macrocirculation, this is not the case in the microcirculation where a large variation in WSS between different vessels has been shown to occur by a number of in vivo studies (Lipowsky et al. 1978; Pries et al. 1990, 1994, 1995a,1995b; Lipowsky 1995; Koutsiaris et al. 2007, 2013). Theoretical works built within the framework of Murray's law but accounting for the non‐Newtonian character of blood have predicted WSS variations in the microcirculation in agreement with in vivo studies (Pries et al. 1990, 1995a,1995b; Zakrzewicz et al. 2002; Sriram et al. 2014a,2014b). These in vivo studies and theoretical models, however, do not provide WSS variation within each vessel itself. In the microcirculation, blood vessels are rarely straight in length. These vessels frequently bifurcate into smaller vessels or merge to form larger vessels, forming microvascular networks whose morphology and architecture are quite complex. As a result, WSS generally cannot be constant along a vessel length. Rather, vascular bifurcations, convergences, and vessel tortuosity, in conjunction with the particulate nature of blood in the microcirculation, are expected to result in 3D WSS variations.

Quantifying such 3D spatial variations is important as they result in WSS gradients (WSSG). ECs are known to respond not only to WSS but also to WSSG (Tardy et al. 1997; Nagel et al. 1999; Ostrowski et al. 2014); such responses are often linked to cell migration, and, hence, they have implications in vascular development and remodeling. WSSG has received some attention in the macrocirculation (e.g., DePaola et al. 1992; LaMack and Friedman 2007; Dolan et al. 2013), but rarely in the microcirculation. Noren et al. (2000) observed spatial gradients in single microvascular bifurcations in vivo, and confirmed by computational modeling. Kim and Sarelius (2003) also observed WSSG in vivo near vessel convergences. Yan et al. (2012) studied the effects of WSSG in vivo and in silico on cell adhesion in curved microvessels. (Liu et al. (2008) demonstrated WSS variations for a single‐phase fluid flowing in curved vessels, while Wang and Bassingthwaighte (2003) predicted such variations using a two‐phase model of blood. Ye et al. (2016) observed asymmetries in WSS due to a single bifurcation in silico using a 2D model. Evidently, there is a large void in quantifying the full 3D variation of WSS and its gradient as they arise in microvascular networks. Thus, our first objective in this study is to provide such a quantification in physiologically similar in silico microvascular networks comprised of multiple bifurcations, convergences, and winding vessels.

In the microcirculation, the cellular character of blood significantly affects the hemodynamics. The most direct hemodynamic effect of the flowing erythrocytes is a blunt velocity profile. Accordingly, WSS and WSSG within each vessel are expected to be affected by the presence of erythrocytes. Using a numerical model of flowing red blood cells (RBCs) in single straight microvessels, Freund and Vermot (2014) showed that the RBCs resulted in fluctuations of WSS. This observation is not possible unless individual RBCs are explicitly modeled. Most in vitro studies dealing with EC response to WSS and WSSG considered an RBC‐free medium (e.g., Nagel et al. 1999; Li et al. 2002; Thi et al. 2004; Li et al. 2005; Kadohama et al. 2007; LaMack and Friedman 2007; Yamamoto and Ando 2013; Ostrowski et al. 2014). The aforementioned numerical studies dealing with WSS and WSSG (Noren et al. 2000; Wang and Bassingthwaighte 2003; Liu et al. 2008; Yan et al. 2012) did not explicitly model individual RBCs. Therefore, there is also a need to quantify the role of RBCs on the distribution of WSS and WSSG in physiological microvascular networks, which is the second objective of this study.

**Figure 1:**
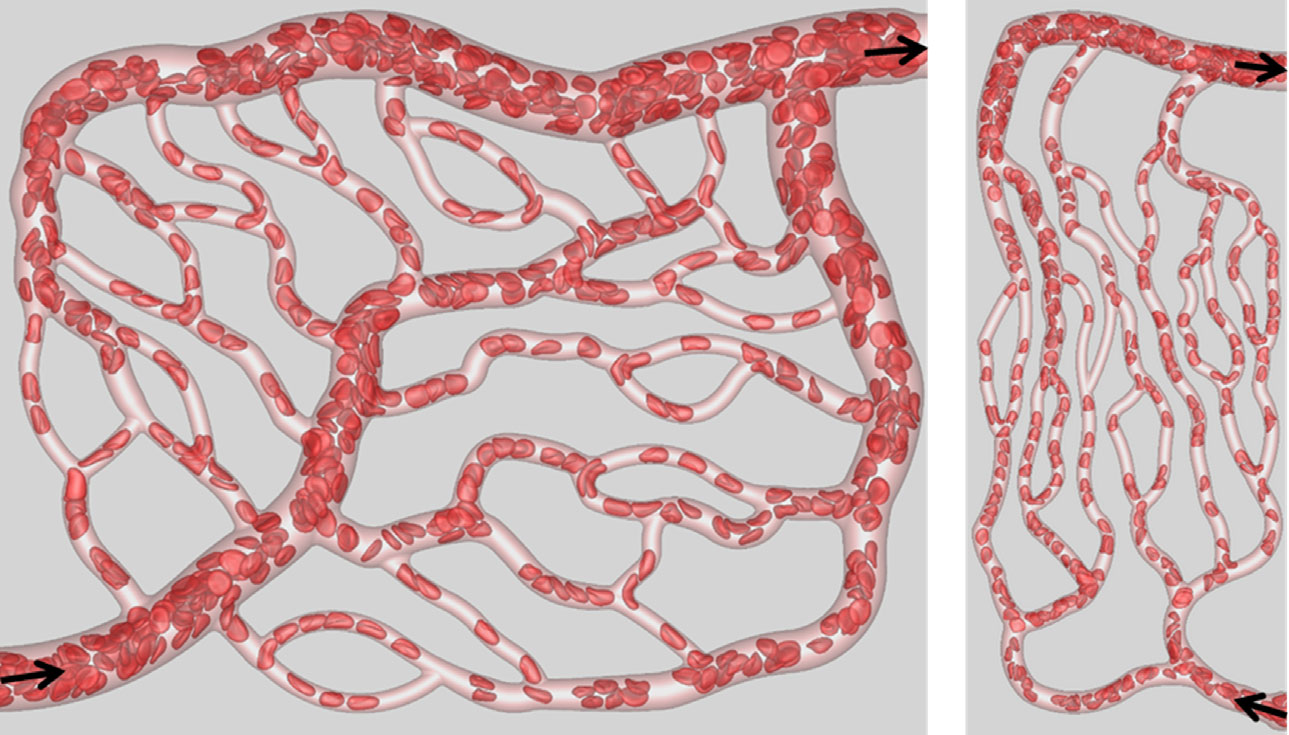
Visualization of RBC flow through two networks used in the simulations. Arrows indicate flow direction.

Recently, we have developed a high‐fidelity, 3D computational model to study blood flow in complex microvascular networks resembling in vivo‐like architectures as shown in Figure 1 (Balogh and Bagchi 2017a,2017b, 2018). The model explicitly considers the deformation of individual RBCs flowing through the networks that are comprised of multiple bifurcations, convergences, and tortuous vessels. Using such simulations, here we present a detailed study on the fully 3D spatial variations of WSS and WSSG in the entire network. By considering two types of simulations, with pure plasma only and in the presence of RBCs, we quantify the influence of RBCs on WSS and WSSG. To our knowledge, this is the first in silico study to provide complete 3D maps of WSS and WSSG in entire in vivo‐like microvasculatures with RBC deformation resolved.

## Methodology

The computational methodology and simulation details are given in (Balogh and Bagchi 2017a,2017b). Three microvascular networks, such as those in Figure 1, were designed in silico following in vivo images and data (Cassot et al. 2006; Benedict et al. 2011). The networks are designed to resemble morphology observed in vivo, and are comprised of multiple bifurcations, mergers, and winding vessels. Collectively, they contain 138 vessels, with diameters ranging from 6 to 24 *μ*m and lengths from 25 to 165 *μ*m, 45 bifurcations, and an equal number of convergences. The average overall path length from inlet to outlet ranges from 500 to 620 *μ*m, and the overall volumes simulated are about 1.6 × 10^6^ *μ*m^3^. The network design utilizes in vivo data in conjunction with Horton's law describing the relationship between vessel diameters at bifurcations and convergences. Three orders of the Strahler scheme span both the arterial and venous sides. The networks are built using a CAD software, and vessel walls are discretized using a triangulated surface mesh. Each network is immersed within a rectangular box that is discretized by a mesh of approximately 8 × 10^7^ grid points. The governing equations for the flow are the unsteady Stokes equations and the continuity equation. The numerical methodology is based on the immersed boundary method (IBM), implemented using a staggered‐grid finite‐volume/spectral approach (Balogh and Bagchi 2017a). The spatial derivatives in the flow equations are evaluated using a second‐order differencing. The time integration is performed by a projection method, where an advection‐diffusion equation is solved using an alternating direction implicit (ADI) scheme, and a Poisson‐type equation is solved to enforce the conservation of mass. For each network, four different simulations were performed using either pressure or flow rate boundary conditions. For the pressure boundary condition, a physiological pressure difference in the range 0.3–1.0 Pa/*μ*m was specified between the inlet and outlet (Lipowsky et al. 1978; Fung 1996). For the other condition, the flow rate in the range (2–4.6) × 10^−13^ m^3^/sec was used in the main feeding artery. The no‐slip condition on the vascular walls is satisfied using a sharp‐interface ghost node IBM (Balogh and Bagchi 2017a).

The deformation of every single RBC flowing through the networks is resolved. Each RBC is modeled as a sac of fluid enclosed by a hyperelastic membrane, and having a biconcave discocyte resting shape with an end‐to‐end distance of 7.8 *μ*m, surface area of 134.1 *μ*m^2^, and volume of 94.1 *μ*m^3^ (Fung 1996). The fluid inside the RBC represents hemoglobin and the fluid outside is plasma. The viscosity of each fluid is taken to be 0.005 and 0.001 Pa‐sec, respectively. The RBC membrane is assumed to possess resistance against shearing, area dilation, and bending. The shearing deformation and area dilation are modeled using the strain energy function developed by Skalak et al. (1973), and the bending resistance is modeled following Zong‐can and Helfrich (1989). The RBC surface is discretized using Delaunay triangles, and a finite‐element method is used to compute the elastic stresses in the membrane (Balogh and Bagchi 2017a). The stresses are coupled to the fluid motion by a continuous forcing IBM (Balogh and Bagchi 2017a). RBCs are initially distributed randomly throughout the networks. As the simulations progress, the distribution naturally develops and results in a heterogeneous distribution of RBCs as observed in vivo. The hematocrit in the main feeding artery is maintained at around 30%. A visualization from the simulations as presented in Figure 1 shows the large deformation of RBCs and a wide range of shapes as observed in vivo, namely, bullet/parachute and slipper shapes, and single‐ or multi‐file motions.

As the simulations progress over time, the hemodynamic quantities evolve. Simulations are run for a physical time of about 0.7 sec, and data are collected every 0.5 msec interval to perform time‐averaging. While the actual WSS varies in space and time, our main focus for the present work is on the time‐averaged WSS. It has been shown (e.g., (Freund and Vermot 2014)) that even in a straight tube the time‐dependent WSS patterns in the presence of RBCs are 3D and asymmetric, while this would not be the case for the time‐averaged WSS under such circumstances. For the present work, the geometric complexity also gives rise to 3D and asymmetric patterns in the time‐averaged WSS. Thus, while we do present some general data on its temporal nature in a subsequent section, our primary focus is the time‐averaged WSS.

The time‐averaged WSS is determined from the time‐averaged velocity field following the approach described in (Freund and Vermot 2014) by computing the traction vector **t** = ***τ*** · **n** at the wall, where ***τ*** is the stress tensor, and ***n*** is the unit normal vector. Because of the irregular geometry of tortuous vessels, bifurcations, and convergences, a coordinate system local to each vertex of the mesh discretizing the walls has to be used. A natural choice is the cylindrical coordinate system with the axial coordinate (*s*) along the direction of the “local” flow close to the wall, and the radial coordinate (*r*) along the local wall‐normal direction. By the no‐slip condition, the components of the traction vector are then expressed as $t_{s}=\mu \partial u_{s}/\partial r$, $t_{r}=-p+2\mu \partial u_{r}/\partial r$, and $t_{\theta }=\mu \partial u_{\theta }/\partial r$, where *θ* represents the circumferential direction. However, our posteriori analysis shows that *t*_*θ*_ and the viscous part of *t*_*r*_ are roughly four to five orders of magnitude less than *t*_*s*_. Thus, only the axial component *t*_*s*_ will be referred to as the WSS and denoted by *τ*. The velocity derivatives are evaluated numerically at each mesh vertex using second‐order differencing. The velocity components at each location in the numerical stencil are interpolated from the simulation velocity, and converted to the local cylindrical coordinate system. The stencil used for the velocity gradient calculations is less than 300 nm, and thus it resides within the cell‐free layer. Hence, the plasma viscosity is used in the above expression for WSS.

To quantify the influence of RBCs on WSS, we also perform simulations involving the flow of plasma only (i.e., without RBCs) in the designed networks using identical boundary conditions. WSS in the presence of RBCs will be denoted by *τ*_RBC_ and that without RBCs will be denoted by *τ*_pl_.

The time‐averaged WSS, *τ*, varies axially and circumferentially throughout the networks. Also of interest is a spatially averaged WSS, $\overset{¯}{\tau }$, averaged over a region of interest (ROI) in a network, that is defined using an area‐weighted average as

$$\overset{¯}{\tau }=\int \;\;\;\int \tau dA/A$$

where *A* is the vascular surface area of the ROI, which could be a vessel, bifurcation, or convergence. As the integration is performed numerically over the surface mesh for the ROI, *dA* refers to the elemental area associated with the specific location of *τ* on the mesh.

WSSG is computed from *τ* using second‐order central differencing in the direction of the specified WSSG component (i.e., axial or circumferential). The *τ* value at each location in the stencil is interpolated from the values at the surrounding surface mesh vertices, with the total distance spanned by the stencil equal to that used for the calculation of *τ* itself.

## Results

### Network level variation of WSS

**Figure 2:**
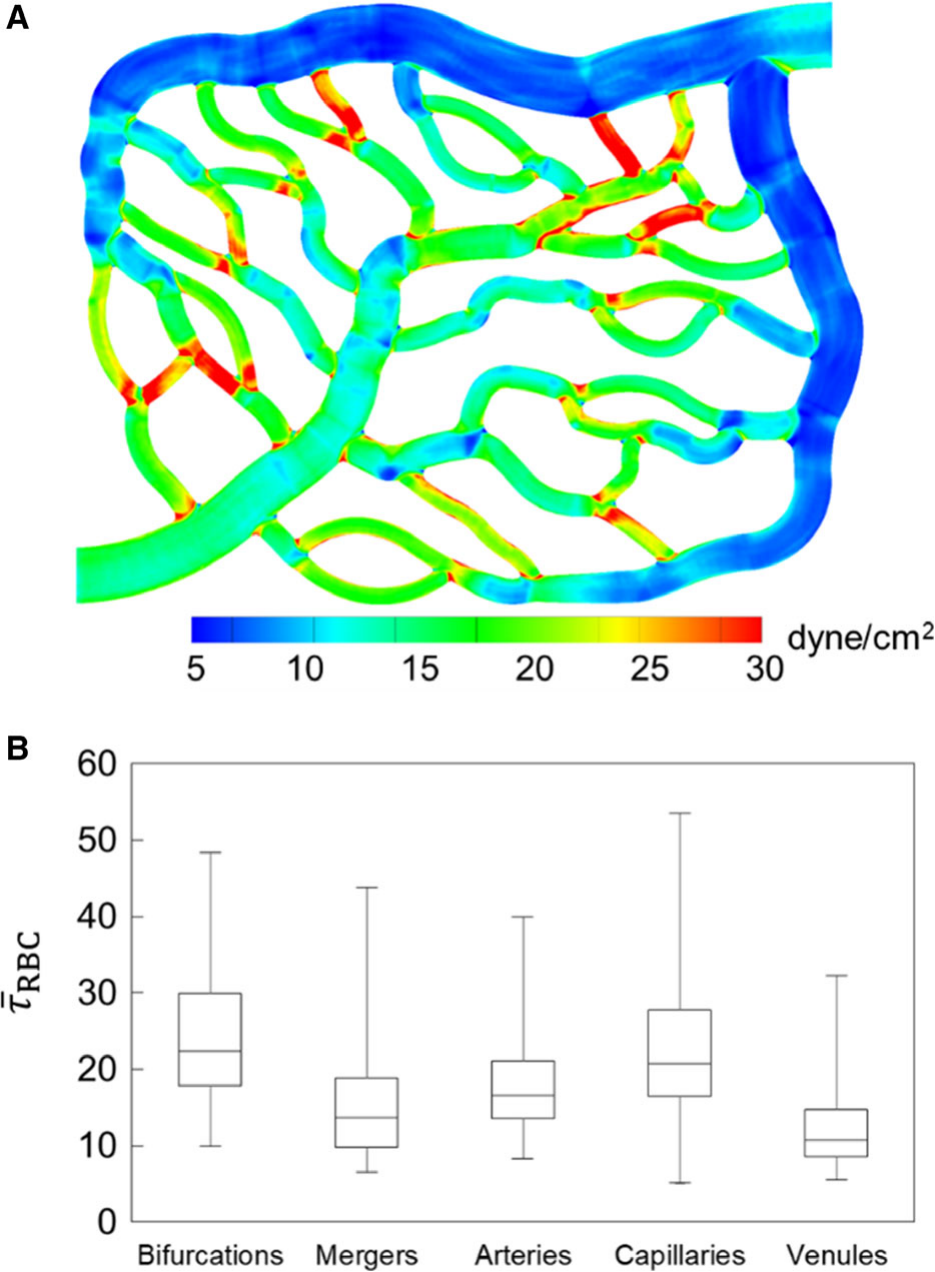
(A) 3D distribution of *τ*_RBC_ in one of the network simulations. (B) ROI‐averaged WSS ${\overset{¯}{\tau }}_{\mathrm{RBC}}$ in different regions based on all simulations, in dyne/cm^2^.

The complete 3D distribution of *τ*_RBC_ is given in Figure 2A as predicted for one of the simulated networks. In agreement with in vivo observations (e.g., Pries et al. 1994, 1995a,1995b; Lipowsky 1995; Koutsiaris et al. 2007, 2013), WSS is seen to be higher in vessels on the arterial side than the venous side, and is the highest in capillaries. Additionally, our data shows that WSS is even higher at capillary bifurcations. Furthermore, a wide variability in WSS is observed from one vessel to another within the same group. Figure 2B shows ${\overset{¯}{\tau }}_{\mathrm{RBC}}$ for different ROIs of the networks, namely, bifurcations, mergers, arterioles, capillaries, and venules. ${\overset{¯}{\tau }}_{\mathrm{RBC}}$ is the highest at bifurcations, followed by capillaries, and is the lowest in venules. In all ROIs, a large range of ${\overset{¯}{\tau }}_{\mathrm{RBC}}$ is observed as implied by the box‐whisker plot, with the maximum range occurring in capillary vessels.

**Figure 3:**
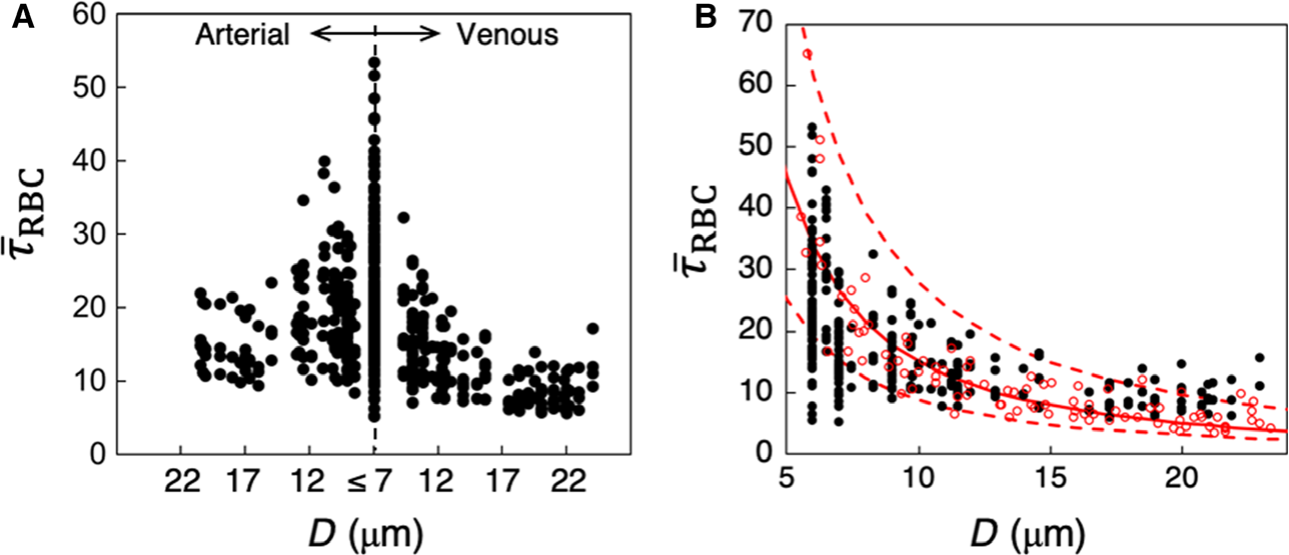
(A) Predicted ${\overset{¯}{\tau }}_{\mathrm{RBC}}$ in all vessels in the simulated networks, in dyne/cm^2^. (B) Comparison of predicted ${\overset{¯}{\tau }}_{\mathrm{RBC}}$ (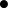) with in vivo data (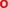) from (Koutsiaris et al. 2007) in capillaries and venules. The red continuous curve is the best fit of the experimental data, and dash curves are the 95% confidence intervals. These curves are presented as reported in (Koutsiaris et al. 2007).

${\overset{¯}{\tau }}_{\mathrm{RBC}}$ for each vessel (capillaries, arterioles, and venules) is presented in Figure 3A as a function of vessel diameter. It is evident that ${\overset{¯}{\tau }}_{\mathrm{RBC}}$ increases with decreasing diameter, in agreement with in vivo observations (Lipowsky et al. 1978; Pries et al. 1994, 1995a,1995b; Koutsiaris et al. 2007). The scatter to the data is also consistent with in vivo observations (Klitzman and Johnson 1982; House and Lipowsky 1987; Pries et al. 1994), and is primarily due to the heterogeneity in hemodynamic quantities such as hematocrit and flow rate, observed across all vessels, which is a hallmark of microvascular blood flow (Pries et al. 1994).

To provide a quantitative validation of the simulations, direct comparisons are made with both in vivo and theoretical values as reported in the literature. Figure 3B gives a comparison of the predicted WSS with in vivo data from (Koutsiaris et al. 2007) as a function of vessel diameter, and shows a good agreement between the two. Additional comparisons can be made against reported in vivo data as follows. In the precapillary arterioles, predicted WSS ranges from approximately 8–40 dyne/cm^2^, which is in agreement with in vivo data reported by Sarelius (1993) who measured values ranging from approximately 5–30 dyne/cm^2^ for arterioles of 13–30 *μ*m diameter. In capillary vessels, our simulations predict ${\overset{¯}{\tau }}_{\mathrm{RBC}}$ ranging from approximately 5–55 dyne/cm^2^, which is also similar to that reported by Lipowsky et al. (1978) in the range 10–50 dyne/cm^2^ in 7 *μ*m diameter vessels in vivo. A few comments should be made regarding comparisons against the in vivo data. At the level of individual vessels, WSS is affected by the vessel hematocrit. The in vivo studies either do not report the vessel hematocrit, or provide the systemic hematocrit only. In the microcirculation, actual hematocrit is less than the systemic one, and it can vary across vessels of the same diameter (Lipowsky et al. 1978; Pries et al. 1994). It is also noted that our model considers rigid vessel walls without any specific treatment for the endothelial surface layer that exists in vivo.

**Figure 4:**
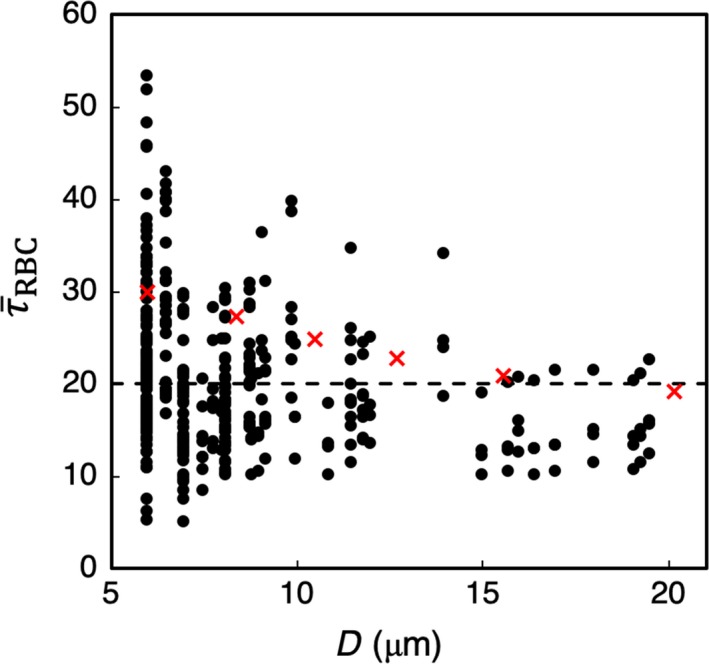
Predicted ${\overset{¯}{\tau }}_{\mathrm{RBC}}$ (dyne/cm^2^) in capillaries and arterioles in the simulated networks, compared with theoretical values. (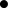) are values from the simulations, (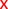) are values reported in (Sriram et al. 2014b) based on their non‐Newtonian model of blood flow in vascular networks, and the (‐‐‐) line gives the constant WSS predicted by Murray's Law.

Comparisons are made with theoretical values reported in the literature in Figure 4. Here, we have plotted the WSS in capillaries and arterioles as predicted by the simulations, along with data reported in (Sriram et al. 2014b) based on their theoretical model of blood flow in vascular networks. This theoretical model was derived based on principles inherent to Murray's Law, but also accounts for the non‐Newtonian behavior of blood in microvessels. Also included in this figure is the constant WSS value predicted by Murray's Law as reported in (Sriram et al. 2014b). As can be seen, the non‐Newtonian model does not capture the data scatter predicted by the simulations and also observed in vivo, but it does give values that are of a similar order of magnitude. Furthermore, it follows a similar trend as the simulation data in that the WSS increases with decreasing vessel diameter, which is also a known in vivo trend in the microcirculation. In comparison with the Murray's Law value shown in this figure, the predicted departure by the simulation data and that of (Sriram et al. 2014b) from this constant‐value trend is in line with, as mentioned, well‐established behavior of blood flow in microvessels. It is interesting to note the average WSS among all vessels is generally of the same order of magnitude as the constant value predicted by Murray's Law. This suggests that at the network level, or in a more aggregate sense, the WSS from the simulations is in general agreement with the optimal work per volume for a vasculature following Murray's Law.

The WSS data in Figures 3B and 4 provides direct comparisons between the simulations and both in vivo data and that computed based on theoretical concepts. Having established that the simulation data are in agreement with these prior works in terms of average WSS values per vessel, subsequent sections investigate and analyze the local 3D spatial variations of the WSS within each vessel that give rise to these predicted average values.

#### Phase separation and WSS heterogeneity

It has been well established in prior works that the disproportionate partitioning of cells relative to the flow at network bifurcations (i.e., phase separation) contributes to a heterogeneous distribution of hemodynamic quantities across microvascular networks (e.g., Popel and Johnson 2005). This behavior has been extensively studied both experimentally and theoretically (Schmid‐Schonbein et al. 1980; Pries et al. 1989; Roberts and Olbricht 2006; Barber et al. 2008; Li et al. 2012; Sherwood et al. 2014; Shen et al. 2016). For the simulations on which the present analysis is based, our prior work (Balogh and Bagchi 2018) investigated various aspects of such cell partitioning within the networks. Relative to the present work, it is of interest to examine the relationship between such phase separation and WSS heterogeneity between vessels.

**Figure 5:**
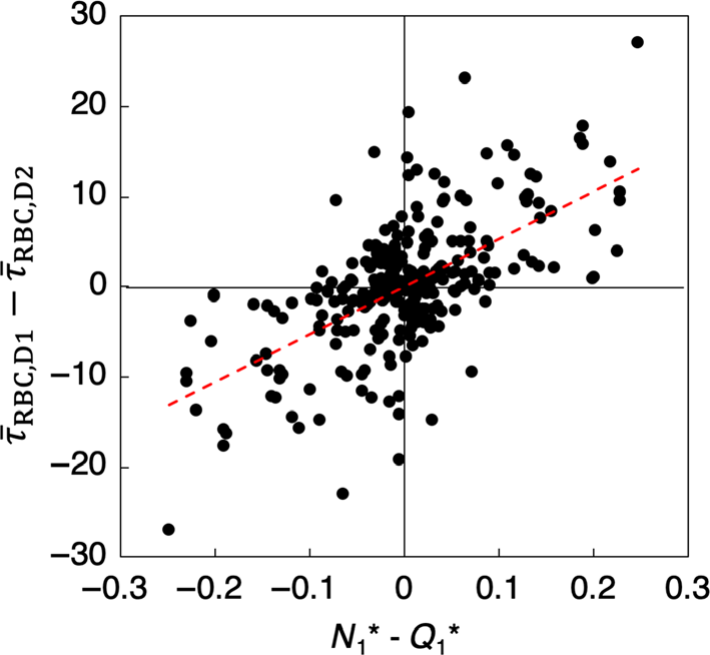
Degree of phase separation (N_1_*―Q_1_*) versus the discrepancy in WSS between daughter vessels, ${\overset{¯}{\tau }}_{\mathrm{RBC},D1}-{\overset{¯}{\tau }}_{\mathrm{RBC},D2}$ (dyne/cm^2^), for all bifurcations. The red dashed line is a linear regression of the data.

Each bifurcation within the simulated networks consists of a feeding vessel that bifurcates into two daughter vessels. We quantify the degree of phase separation at each bifurcation by first computing the flow ratio (Q*) and RBC flux ratio (N*) between each daughter branch (*D*_1,2_) and the feeding vessel (*F*). For daughter vessel *D*_1_, the flow ratio is defined as *Q*_1_* = *Q*_*D*1_/*Q*_*F*_ and the RBC flux ratio is defined as *N*_1_* = *N*_*D*1_/*N*_*F*_, and similarly for daughter vessel *D*_2_. The means by which these quantities are computed from the simulation data are detailed in (Balogh and Bagchi 2018). The degree of phase separation is then quantified by N_1_*―Q_1_*, which gives the degree of disproportionality in the cell‐flow partitioning at the bifurcation. This quantity is plotted in Figure 5 for each daughter vessel versus the difference in WSS between daughter vessels, that is, ${\overset{¯}{\tau }}_{\mathrm{RBC},D1}-{\overset{¯}{\tau }}_{\mathrm{RBC},D2}$.

The general trend shown in Figure 5 is that the magnitude of the WSS discrepancy between daughter branches increases with increasing magnitude of phase separation. Thus, as the degree of phase separation increases, so does the heterogeneity in WSS between daughter branches. While such behavior is expected from the known connection between phase separation and heterogeneity in hemodynamic quantities, this provides a quantitative link to network‐level WSS heterogeneity in the present simulations.

### Cellular influence on WSS

The influence of RBCs on WSS is isolated by comparing the results based on the simulations with RBCs to that without RBCs, that is, the flow of plasma only. An important consideration in comparing simulations with and without RBCs is the choice of boundary condition (BC). As discussed in §2, the boundary conditions used are either flow rate specified or pressure specified. With the pressure BC, the resulting pressure drops in vessels in a network yield similar values in RBC and plasma‐only simulations, while the flow rates are different. As such, the influence of RBCs is manifested in differences in flow rates. With the flow rate BC, pressure drops in vessels become different for RBC and plasma‐only simulations, resulting also in different shear stresses. Thus, to appropriately quantify the influence of RBCs on WSS, only the flow BC simulations are considered.

**Figure 6:**
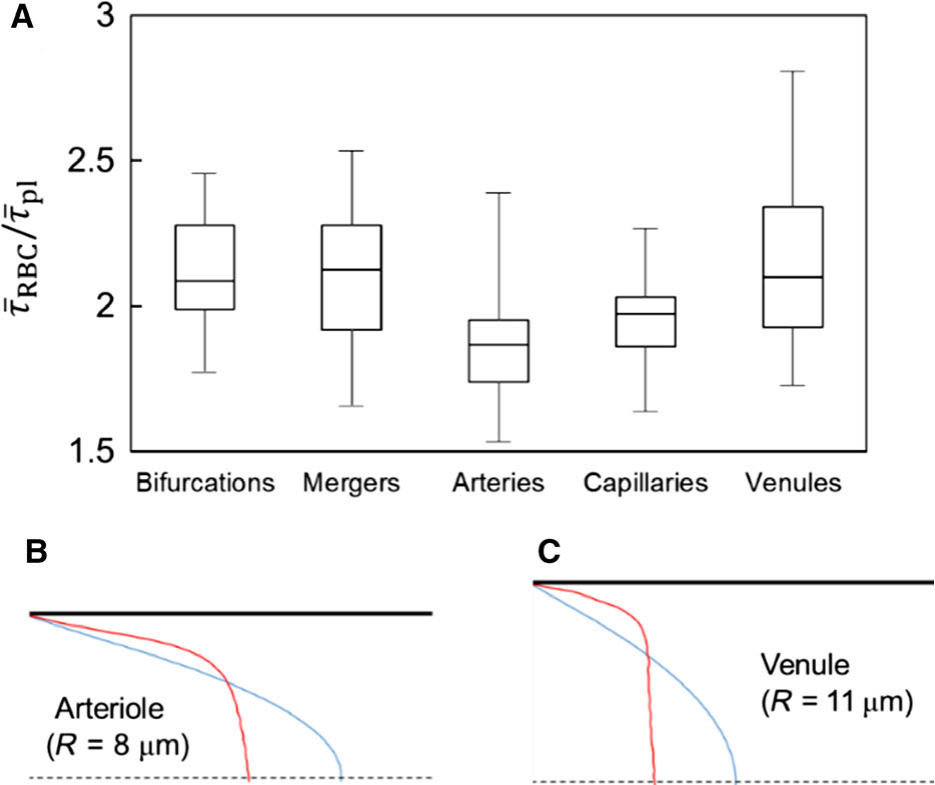
(A) Influence of RBCs on WSS is quantified by the ratio ${\overset{¯}{\tau }}_{\mathrm{RBC}}/{\overset{¯}{\tau }}_{\mathrm{pl}}$ in different ROIs. (B) & (C): Explanation of greater RBC influence on the venous side. Velocity profiles in the vessels of same order (order 3) are shown for the arterial side (B), and venous side (C). Red: velocity profile from RBC simulation. Blue: profile from plasma‐only simulation.

The influence of RBCs on WSS is quantified by the ratio ${\overset{¯}{\tau }}_{\mathrm{RBC}}/{\overset{¯}{\tau }}_{\mathrm{pl}}$, which is shown in Figure 6A for different ROIs. It is seen that RBCs cause an increase in WSS in every region. This is expected due to the blunt velocity profile that occurs in the presence of RBCs and which results in a higher velocity gradient at the walls compared to that without RBCs. What is interesting, however, is that the increase is not uniform across the vasculature: significantly higher values of ${\overset{¯}{\tau }}_{\mathrm{RBC}}/{\overset{¯}{\tau }}_{\mathrm{pl}}$ are observed in bifurcations, mergers, and venules, with the highest occurring in venules. The lowest value occurs in arterioles. This is particularly noteworthy because ${\overset{¯}{\tau }}_{\mathrm{RBC}}$ itself is the lowest on the venular side and at mergers as noted previously in Figure 2. Thus, the influence of RBCs on WSS is higher at ROIs where WSS itself is relatively lower.

In terms of network level WSS trends, the relatively higher increase of WSS on the venous side compared to the arteriolar side is due to the differences in velocity profiles as shown in Figure 6B–C. Vessels of the same order on the venous side have larger diameters than those on the arterial side. Since the flow rate is preserved, this results in a larger difference in the near‐wall velocity gradient between the RBC simulations and plasma‐only simulations on the venous side than on the arterial side.

Subnetwork level WSS trends within different ROIs, including the mechanisms underlying increased RBC influence, are related to local RBC dynamics and interaction with the complex geometry, and are considered in later sections.

### WSS gradient

**Figure 7:**
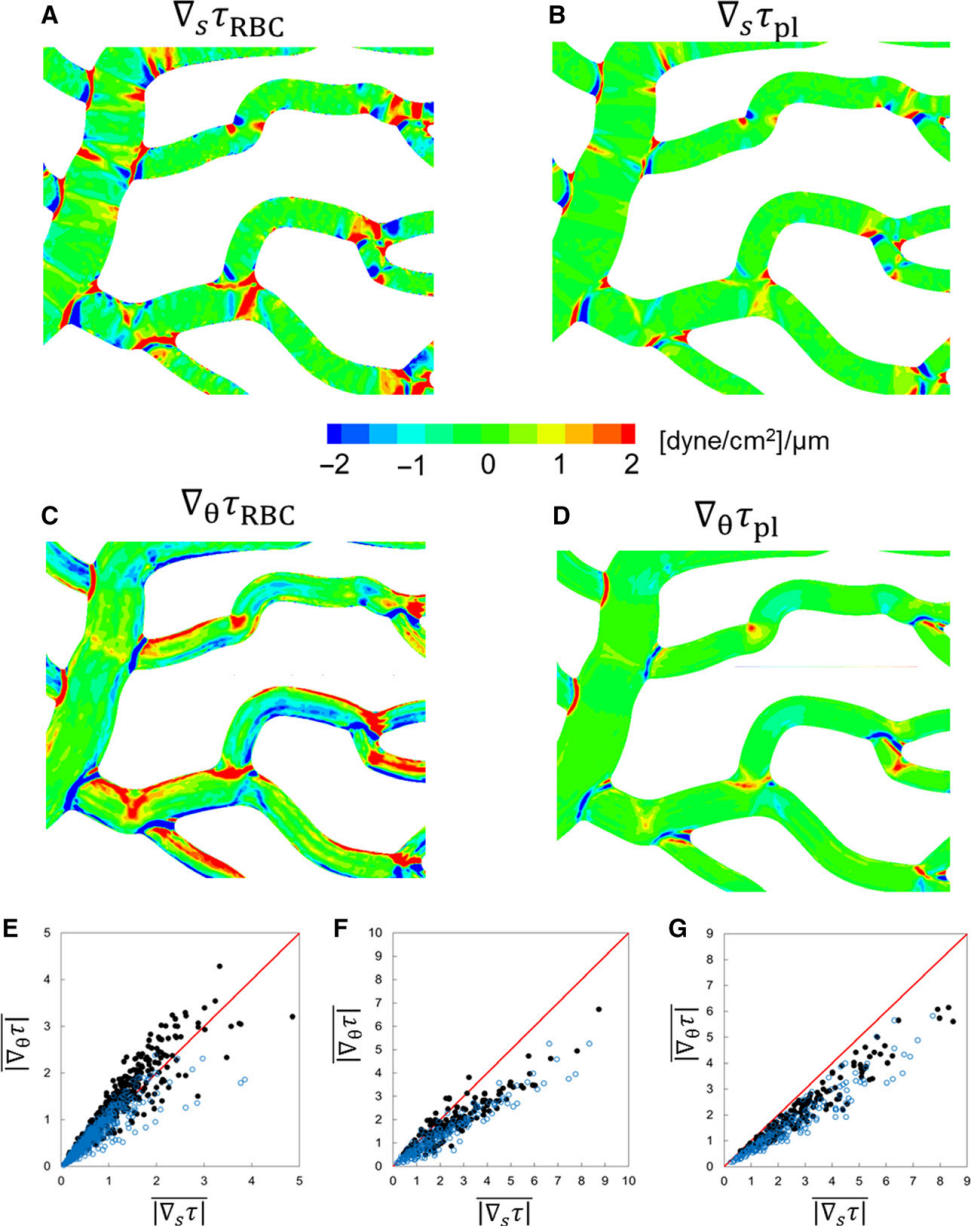
(A)–(D) 3D distribution of WSSG components in a selected region of a simulated network for comparing the RBC and plasma‐only simulations. (E)–(G) Comparison of axial versus circumferential components of WSSG in different ROIs: vessels (E), bifurcations (F), and convergences (G). Simulations with RBCs (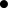); plasma‐only (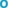). Units are in dyne/cm^2^/*μ*m.

**Figure 8:**
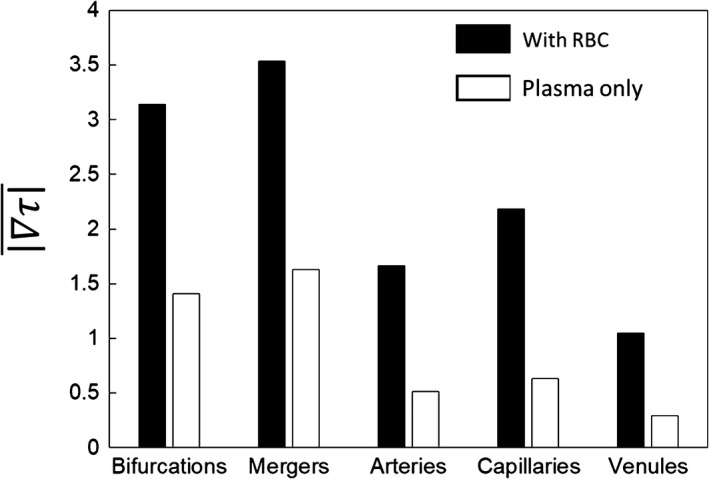
WSSG magnitudes (dyne/cm^2^/*μ*m) in different ROIs in simulations with RBCs (black filled) and without RBCs (unfilled).

A closer inspection of Figure 2A reveals large spatial gradients in WSS throughout the network. To quantify the 3D nature of WSS gradients (WSSG), we compute the axial gradient ∇_*s*_ *τ* and the circumferential gradient ∇_*θ*_ *τ*, which are shown in Figure 7 for a portion of the network for both RBC and plasma‐only simulations. The magnitude of WSSG, $\left|\nabla \tau \right|$, in the two simulations are compared in Figure 8.

A significantly higher WSSG occurs in the presence of RBCs. WSSG magnitudes are significantly higher at bifurcations and mergers, and are lowest in the venules. In addition, high WSSG occurs over wider regions compared to that in the plasma‐only simulations. Figure 7 further shows that both the axial and circumferential gradients increase in the presence of RBCs. However, the increase in the circumferential component is more than that in the axial component. The axial component is primarily dictated by the geometry. As such, for the plasma‐only simulations the axial component is greater than the circumferential component nearly in every region of the network (Fig. 7E–G). For the RBC simulations, the axial component is larger than the circumferential component only at bifurcations and mergers (Fig. 7F and G). On the contrary, in more than 83% of the vessels, the circumferential component is greater than the axial component in the presence of RBCs (Fig. 7E). The details underlying these observed WSSG trends specific to different ROI are discussed in later sections.

### WSS and WSSG temporal variations

**Figure 9:**
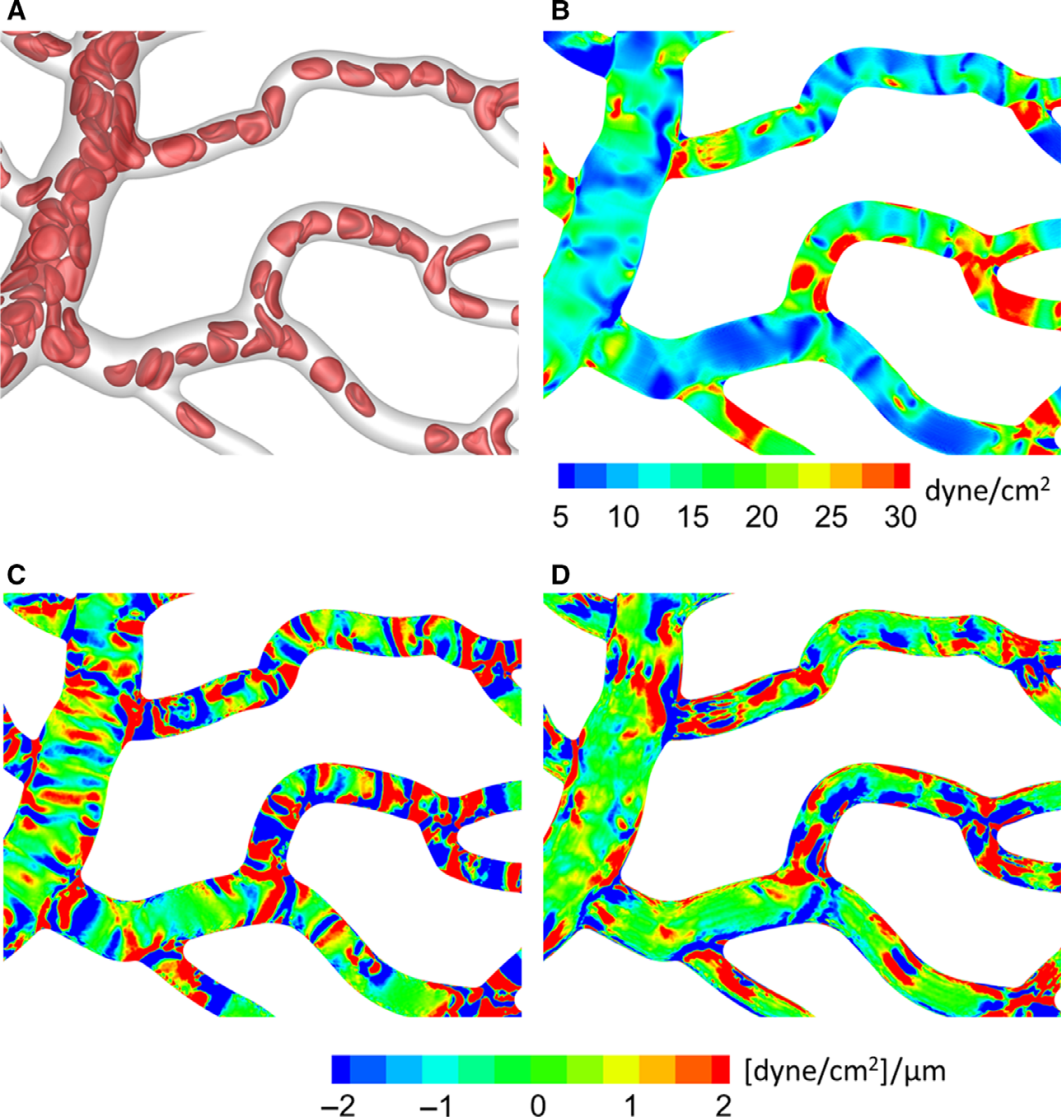
Instantaneous WSS and WSSG spatial variations. (A) Snapshot of RBCs at a specific instant in time. Corresponding instantaneous contours of WSS (B), axial WSSG (C), and circumferential WSSG (D).

As mentioned earlier, the WSS and WSSG vary throughout the networks in both space and time. For the present simulations the temporal variations arise due to the presence of the RBCs. Figure 9 illustrates the instantaneous contours of WSS and WSSG as calculated for a representative section of one network. A snapshot of the RBCs at the given instant in time is shown in Figure 9A, while the corresponding spatial variations in WSS and WSSG at the same instant are given in Figure 9B–D. By comparing Figure 9A and B, the so‐called footprint (Freund and Vermot 2014) of the RBCs on local patterns in the WSS can be generally observed relative to the locations of the cells. Figure 9C and D give the corresponding gradients that result from these instantaneous spatial patterns in Figure B. As the RBCs subsequently flow through the networks these WSS and WSSG patterns migrate with them and evolve, resulting in temporal fluctuations.

Instantaneous contours for the axial gradient (Fig. 9C) show band‐like patterns that form generally oriented transverse to the flow direction, and these move along vessel lengths following the RBCs. Contours for the circumferential gradient (Fig. 9D) also follow the RBCs, but tend to form bands more oriented in the flow direction. Relative to the time‐averaged WSSG trends presented in Figure 7 and the associated discussion, this temporal behavior here is generally conducive to the time‐averaged circumferential WSSG being greater than the axial WSSG as discussed in Section 3.3. That is, for example, over a period during which an RBC passes by a particular point on a vessel, the axial WSSG temporarily increases to a large positive value as the RBC approaches, and then decreases to a large negative value when the RBCs leaves. In contrast, the circumferential WSSG at that same point generally increases to a large value and then decreases to a smaller value of the same sign.

**Figure 10:**
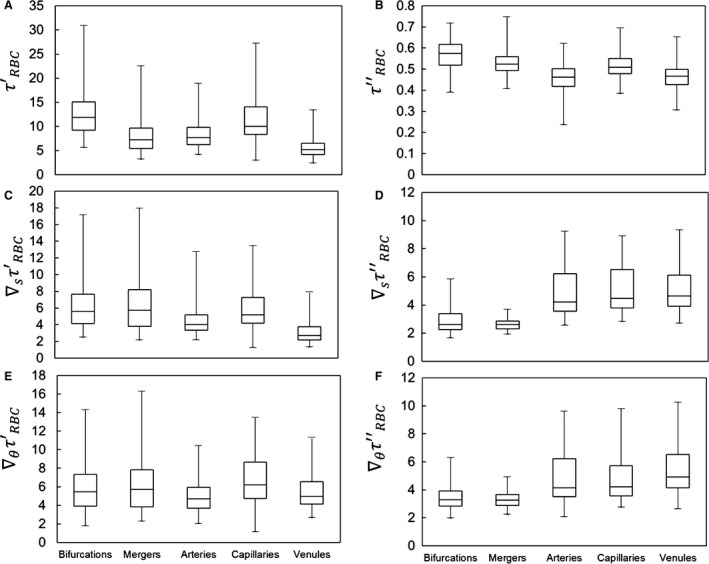
Statistics on absolute and relative RMS values quantifying WSS and WSSG fluctuations in different ROIs, based on data points for all simulations. (A,C,E) give absolute RMS values for *τ*′_RBC_, ∇_*s*_ *τ*′_RBC_, and ∇_*θ*_ *τ*′_RBC_, respectively. (B,D,E) give relative RMS values for *τ*″_RBC_, ∇_*s*_ *τ*″_RBC_, and ∇_*θ*_ *τ*″_RBC_, respectively. Absolute WSS values are in dyne/cm^2^, while absolute WSSG values are in dyne/cm^2^/*μ*m.

To generally quantify the temporal character of the WSS and WSSG, we compute the root‐mean‐square (RMS) of the fluctuations within each ROI. RMS values are first calculated at each vertex of the mesh comprising the network walls based on the time‐dependent signal for WSS or WSSG at the vertex. The RMS value is then determined for each ROI by taking the average of that computed for all vertices associated with it. This is referred to here as the absolute RMS, and is denoted by *τ*′_RBC_, ∇_*s*_ *τ*′_RBC_, and ∇_*θ*_ *τ*′_RBC_ for the WSS and WSSG components, respectively. For the relative RMS, the value at each vertex is scaled by the time‐averaged value for the ROI in which the vertex resides. The value for each ROI is determined by taking the average of that computed for all vertices associated with it, and these are denoted by *τ*″_RBC_, ∇_*s*_ *τ*″_RBC_, and ∇_*θ*_ *τ*″_RBC_. The trends associated with the data points for each of these values from all simulations are presented in Figure 10.

For the WSS, Figure 10A shows that the magnitude of the temporal fluctuations is highest within bifurcations and in capillary vessels, and that these fluctuation magnitudes can be upwards of 30 dyne/cm^2^. Relative to the mean values, Figure 10B shows that fluctuations in the WSS among all ROIs tend to vary between 30 and 75% of the average WSS for the individual ROI, and that these fluctuations tend to be highest within bifurcations and mergers. For the WSSG, Figure 10C and E show that the magnitude of the temporal fluctuations is highest in the bifurcations and mergers, and spans a wider range than in vessels. Interestingly, Figure 10D and F show that when considered relative to the mean values the gradients fluctuate significantly more than the WSS itself, and that the degree of fluctuations span a wider range. The magnitude of the relative temporal fluctuations in the gradients are of a similar order of magnitude between the axial and circumferential components, while the average for each ROI is slightly higher for the circumferential component. These figures also show that the magnitude of the gradient fluctuations ranges anywhere from 2 to 10 times the average for each individual ROI.

Furthermore, contrary to the WSS itself, when considered relative to mean values the WSSG fluctuates more significantly within vessels than within the bifurcations and mergers. This happens because the gradients tend to remain elevated within bifurcations and mergers for longer periods of time, whereas within vessels the gradients are in more of a constant state of flux. That is, as cells enter a bifurcation or merger region they tend to slow down relative to the speed with which they traversed the associated vessel. This leads to the frequency of WSSG fluctuations being higher in vessels than bifurcations and mergers. For the WSS, however, this type of behavior is less pronounced as the WSSG gradients change more sharply than the WSS itself.

### WSS variation at subnetwork levels

We now consider WSS variations in individual segments (ROIs) of the networks, namely, along vessels lengths, and in bifurcations and convergences.

#### WSS variation along vessel length

**Figure 11:**
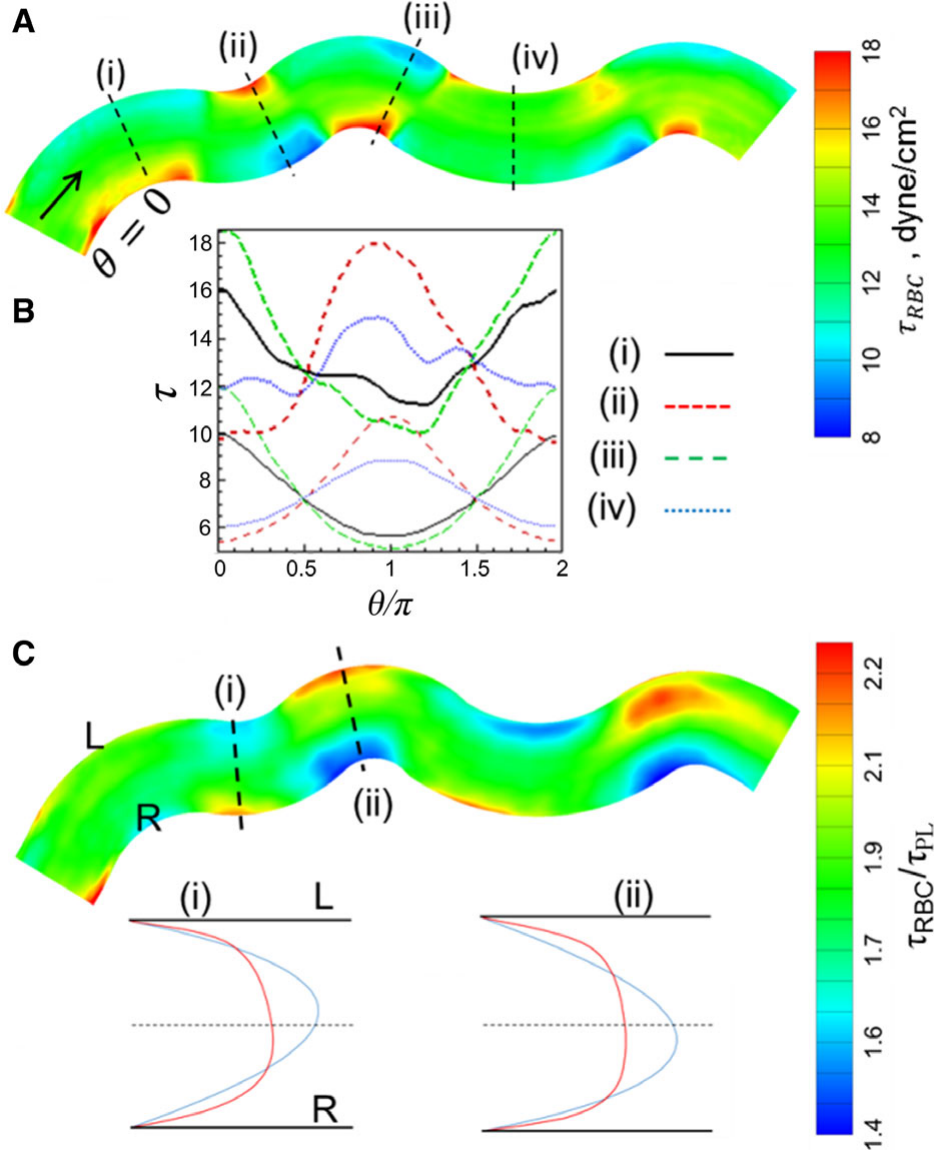
(A) *τ* distribution in a representative tortuous vessel selected from a simulated network. (B) Circumferential distribution of *τ* at four selected locations as shown in (A). Thick curves are for simulations with RBCs, thin curves are without RBCs. (C) Ratio *τ*/*τ*_pl_. Also shown are the predicted velocity profiles at two locations in the vessel for simulations with RBCs (red curve) and without (blue curve). L and R means left and right sides of the vessel looking downstream in the flow direction.

WSS variations along a vessel length arise primarily due to the two geometric effects, namely, vessel bending, and the influence of an upstream bifurcation or merger. They also arise due to the presence of RBCs. Figure 11A shows the variations in WSS profile in a tortuous vessel from a simulated network. It is seen that WSS is higher on the side of the vessel that has the higher curvature. A similar pattern of WSS is also observed in the plasma‐only simulations, although the magnitudes are substantially reduced. Such 3D patterns in WSS arise because the velocity profile in a curved vessel is not axisymmetric; rather it is shifted to the side of the vessel that has the higher curvature. It may be noted that no secondary flow exists in these microvessels. The type of skewness in velocity profiles observed here is a characteristic of flows with negligible inertia, and is different from what would occur in large vessels where secondary flow may cause the velocity profile to shift to the opposite side (Chadwick 1985; Wang and Bassingthwaighte 2003; Verkaik et al. 2009). For a vessel that bends frequently, the maximum WSS switches from one side to the other. This results not only in the variations of WSS along the axial direction but also along the circumferential direction (Fig. 11B). Both axial and circumferential components of WSSG follow the vessel curvature, with the former being highly localized at regions of curvature change, and the latter component being more spread out and diffuse (Fig. 11B). Both components are enhanced in the presence of RBCs, with the circumferential component being more enhanced than the axial one.

The RBC influence on WSS in this tortuous vessel is shown in Figure 11C, where the ratio *τ*_RBC_/*τ*_pl_ is plotted. While *τ*_RBC_/*τ*_pl_ > 1 throughout the vessel, its spatial variation is significant and noteworthy. In fact, the spatial patterns of *τ*_RBC_ and *τ*_RBC_/*τ*_pl_ show opposite trends. Specifically, *τ*_RBC_/*τ*_pl_ is observed to be higher on the side of the vessel that has the smaller curvature, while WSS itself is higher on the opposite side. Thus, RBCs have a greater influence on the side of the vessel where the WSS itself is smaller. This also results in WSSG being more spread out over a larger region in the presence of RBCs, as noted earlier. The reason why the RBC influence is greater on the side of smaller WSS can be understood by comparing the predicted velocity profiles for the simulations with and without RBCs as shown in Figure 11C. The discrepancy in the near‐wall velocity gradients in the two profiles is higher on the side of the vessel that has the smaller curvature. This happens because the velocity profile of pure plasma becomes more skewed to that side compared to the profile in the presence of RBCs. As such, the skewness in the plasma velocity profile oscillates along vessel length to a greater degree than the whole blood velocity profile in response to the changing vessel curvature, resulting in a greater disparity between the near‐wall gradients.

**Figure 12:**
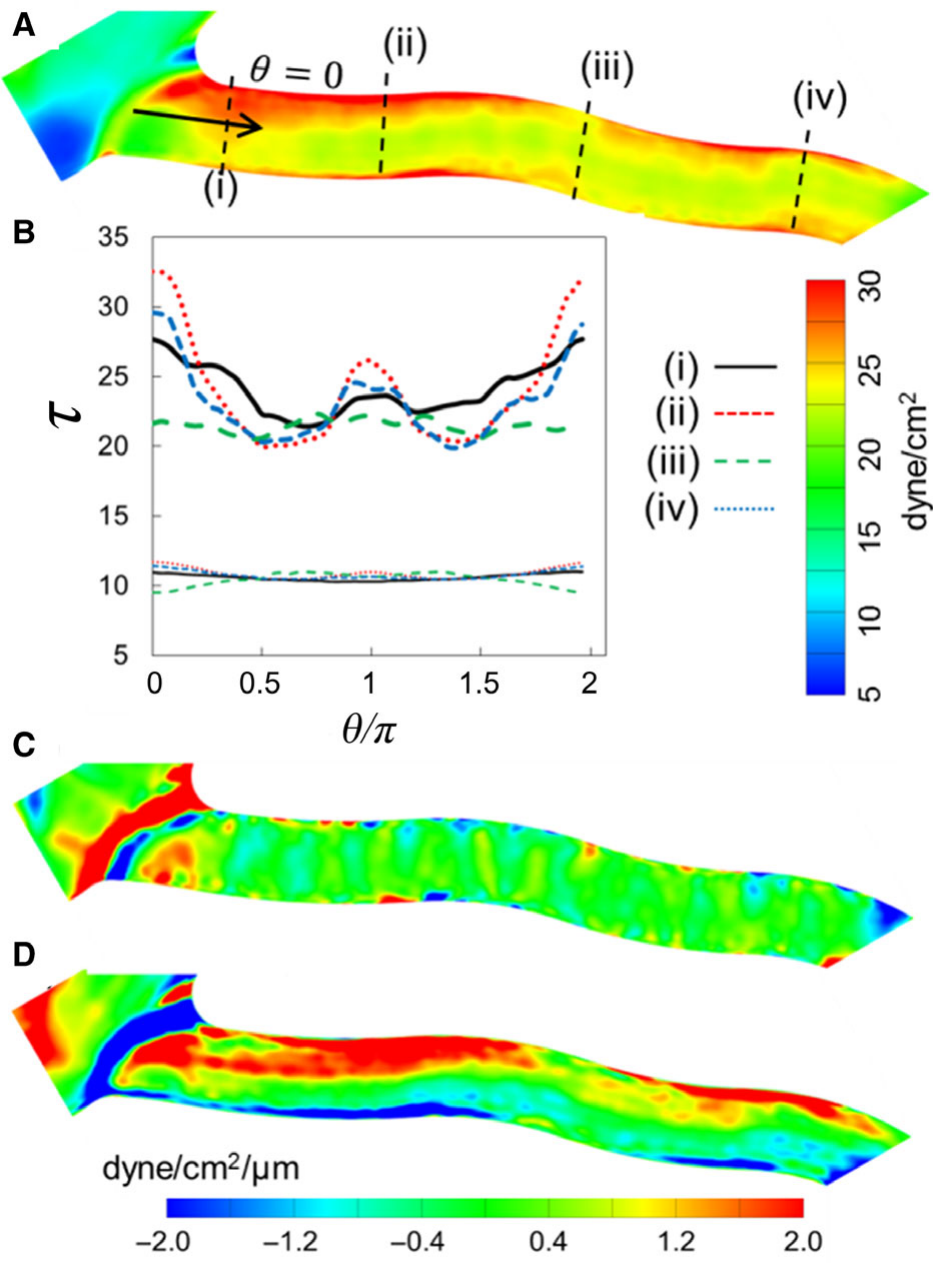
(A) *τ*_RBC_ distribution in a relatively straight vessel. (B) Circumferential distribution of *τ* at four locations along the vessel. Thick curves are for simulations with RBCs, and thin curves are without RBCs. (C) and (D) Axial and circumferential components of WSSG.

We now consider WSS variations in a relatively straight vessel. In such a vessel, the upstream bifurcation or convergence, along with the flow dynamics of RBCs, can cause a variation of WSS as shown in Figure 12A. The presence of an upstream bifurcation creates a WSS profile that varies significantly in the circumferential direction at the inlet to the vessel. This is because as RBCs flow through the bifurcation and enter the vessel, they tend to flow closer to the side of the vessel that is nearest to the apex of the bifurcation (*θ* = 0). As a result, the RBC‐core is shifted toward this side of the vessel and the cell‐free layer is reduced. This, in turn, causes an increased near‐wall velocity gradient, and hence, a higher WSS. If the vessel is relatively straight, the higher WSS on this side persists for a distance along the vessel length. It also results in a significantly higher circumferential WSSG component than the axial component (Fig. 12C–D). In contrast, a circumferential variation of WSS in the same vessel is nearly absent in the plasma‐only simulation (Fig. 12B).

**Figure 13:**
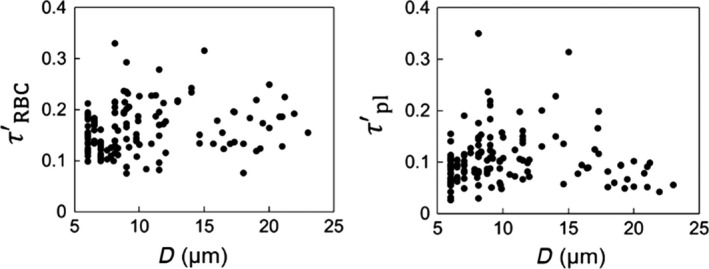
WSS standard deviations in vessels for simulations with RBCs ($\tau_{\mathrm{RBC}}^{\prime }$) and without RBCs ($\tau_{\mathrm{pl}}^{\prime }$).

The above results show that the presence of RBCs not only increases WSS magnitudes but also causes a significantly larger variation in WSS over the vessel length than what would occur in the absence of RBCs. This degree of variation is quantified by the standard deviation *τ*′ of WSS for each vessel, defined as:

$$\tau^{\prime }=\frac{1}{\overset{¯}{\tau }}\sqrt{\frac{\sum_{i=1}^{N}{\left(\tau_{i}-\overset{¯}{\tau }\right)}^{2}}{N-1}}$$

where *τ*_*i*_ is the WSS at vertex *i* of the vessel surface mesh, *N* is the total number of vertices for the vessel, and $\overset{¯}{\tau }$ is the average WSS for the vessel. Figure 13 plots $\tau_{\mathrm{RBC}}^{\prime }$ and $\tau_{\mathrm{pl}}^{\prime }$ versus vessel diameter. In more than 90% of the vessels, $\tau_{\mathrm{RBC}}^{\prime }>\tau_{\mathrm{pl}}^{\prime }$. The average value of $\tau_{\mathrm{RBC}}^{\prime }/\tau_{\mathrm{pl}}^{\prime }$ over all vessels is approximately 2, while in the smallest capillaries its value could be as high as 5. On the whole, this data quantitatively shows that a significantly larger spatial variation of WSS occurs along the vessel lengths in the presence of RBCs.

#### WSS at bifurcations

**Figure 14:**
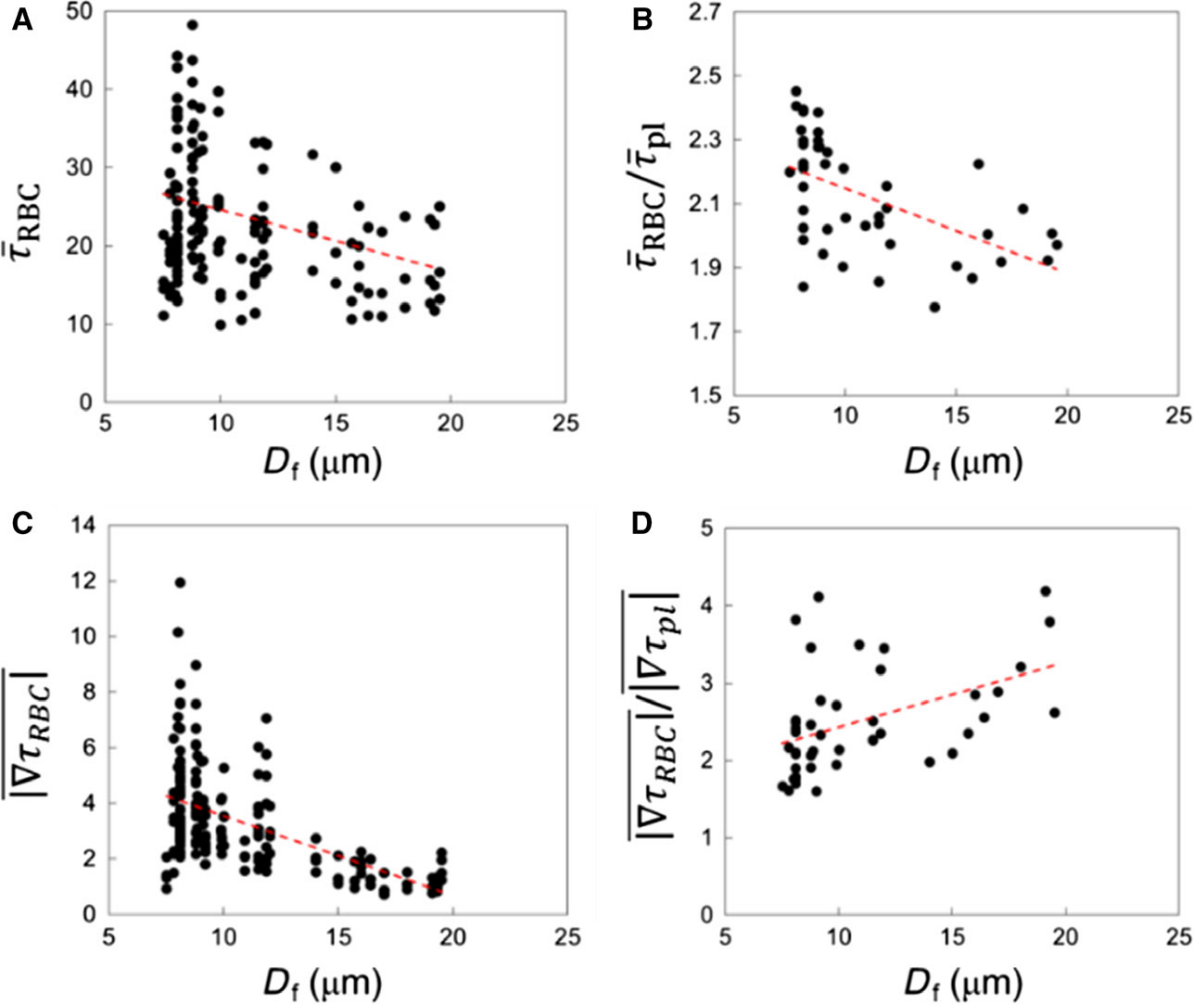
ROI‐averaged data for bifurcation regions. (A) ${\overset{¯}{\tau }}_{\mathrm{RBC}}$, (B) ${\overset{¯}{\tau }}_{\mathrm{RBC}}/{\overset{¯}{\tau }}_{\mathrm{pl}}$, (C) WSSG magnitudes in the presence of RBCs, and (D) ratio of WSSG magnitudes for simulations with and without RBCs, as functions of feeding vessel diameter. Red lines represent a linear regression.

While ROI‐averaged WSS values are observed to be the highest in bifurcations (Fig. 2), there is a wide distribution among different bifurcations. This is shown in Figure 14A, where ${\overset{¯}{\tau }}_{\mathrm{RBC}}$ is plotted for all bifurcations as a function of the diameter (*D*_*f*_) of the respective feeding vessels. Despite scatter in the data, which arises due to the heterogeneity of hemodynamic quantities across the vasculature, ${\overset{¯}{\tau }}_{\mathrm{RBC}}$ tends to increase with decreasing *D*_*f*_. Furthermore, the ratio ${\overset{¯}{\tau }}_{\mathrm{RBC}}/{\overset{¯}{\tau }}_{\mathrm{pl}}$ in Figure 14B also shows an increasing trend with decreasing *D*_*f*_, implying an increasing influence of RBCs in the bifurcations leading to the lowest order capillary vessels. WSSG magnitudes for bifurcations are plotted in Figure 14C, and the ratio $\bar{\left|\nabla \tau_{\mathrm{RBC}}\right|}/\bar{\left|\nabla \tau_{\mathrm{pl}}\right|}$ is shown in Figure 14D. $\bar{\left|\nabla \tau_{\mathrm{RBC}}\right|}$ is observed to increase with decreasing *D*_*f*_ due to increasing cellular influence in capillary bifurcations, while $\bar{\left|\nabla \tau_{\mathrm{RBC}}\right|}/\bar{\left|\nabla \tau_{\mathrm{pl}}\right|}$ shows a weakly decreasing trend because $\bar{\left|\nabla \tau_{\mathrm{pl}}\right|}$ becomes very small as *D*_*f*_ increases.

**Figure 15:**
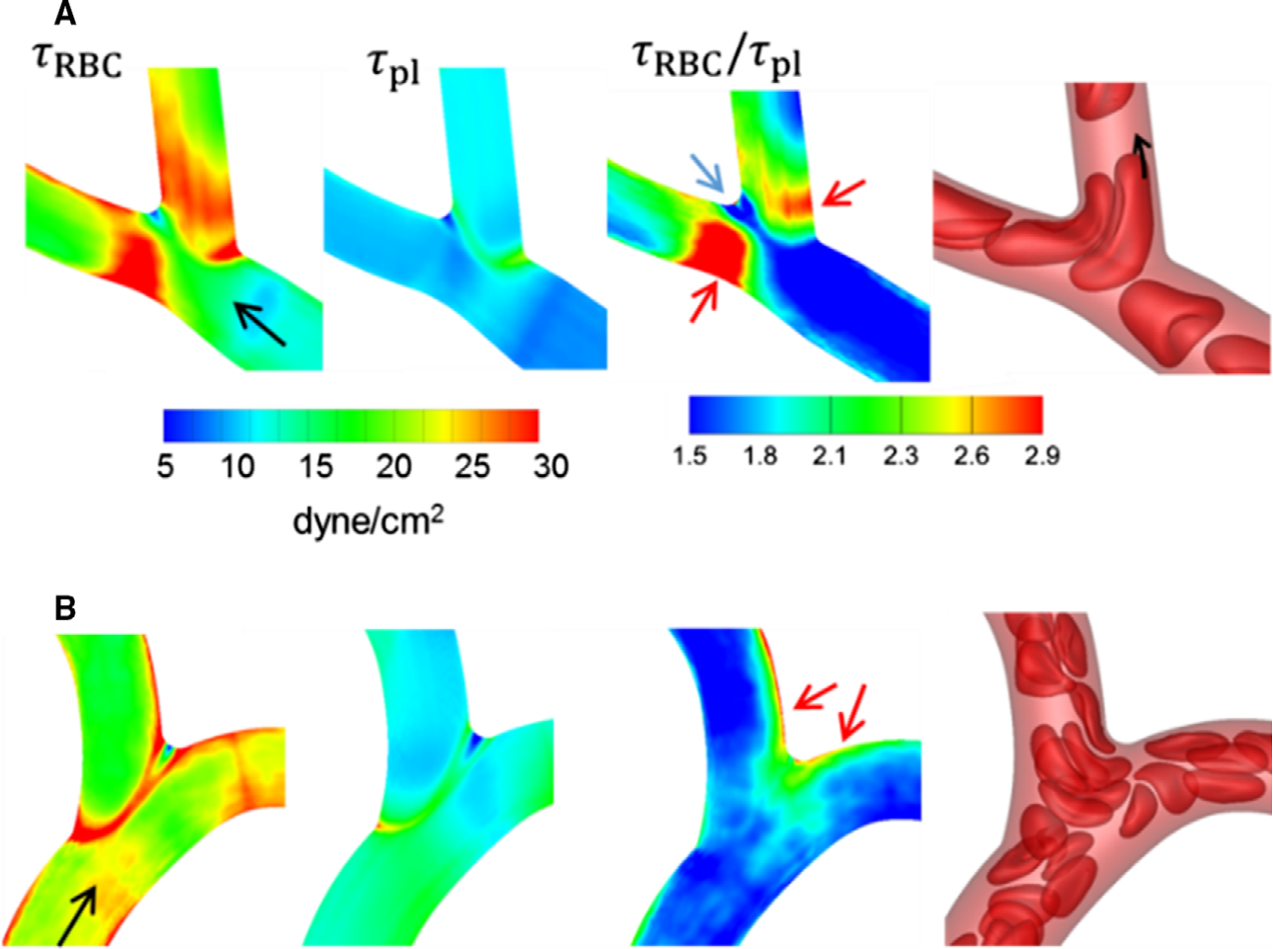
Distribution of WSS at (A) a bifurcation with a relatively smaller feeding vessel (*D*_f_ = 8 *μ*m), and (B) a bifurcation with a larger feeding vessel (*D*_f_ = 14 *μ*m). Shown here are WSS with and without RBCs, their ratio, and snapshots showing RBC flow dynamics. Black arrows indicate flow direction, red arrows indicate locations of the highest WSS ratio, and blue arrow indicates location of the lowest WSS ratio.

We now illustrate the detailed spatial patterns of WSS and their underlying mechanisms at representative bifurcations selected from the networks. A bifurcation with a relatively smaller feeding vessel (*D*_*f*_ = 8 *μ*m) is shown in Figure 15A. Both geometric and cellular influences cause increased WSS at bifurcations. As seen in this figure, the lowest WSS occurs near the apex of the bifurcation. In the presence of RBCs, WSS increases significantly near the entrance to the daughter vessels. This is because of the increased confinement as RBCs squeeze through the smaller diameter daughter vessels. This is the primary mechanism by which WSS increases at capillary bifurcations. The ratio *τ*_*RBC*_/*τ*_*pl*_ plotted in Figure 15A further shows that a spatially heterogeneous influence of RBCs exists even within the bifurcation region. *τ*_*RBC*_/*τ*_*pl*_ is the lowest near the apex, and also relatively smaller along the sides of the daughter vessels that are closer to the apex. In contrast, it is highest along the opposite sides. This occurs because of the RBC dynamics at the bifurcation. Individual RBCs often tend to significantly slow down and straddle around the apex partially blocking the entrance to the daughter vessels. RBCs upstream then flow around the straddling RBC and closer to the opposite sides away from the apex, thereby enhancing the local WSS there.

A bifurcation with a relatively larger feeding vessel (*D*_*f*_ = 14 *μ*m) is considered in Figure 15B. Because of the geometry, WSS increases significantly in a narrow region from where the side vessel emanates. WSS also increases along the sides of both daughter vessels that are closer to the apex. The ratio *τ*_*RBC*_/*τ*_*pl*_ is observed to be higher around the apex and along the sides that are closer to the apex. This trend is opposite to what was observed in Figure 15A for the smaller *D*_*f*_. Such a trend occurs in relatively large diameter bifurcations due to less confinement. In these, RBCs mostly flow along the sides closer to the apex, causing a relatively larger cell‐free layer, and a reduced velocity gradient, on the opposite sides of the vessels.

**Figure 16:**
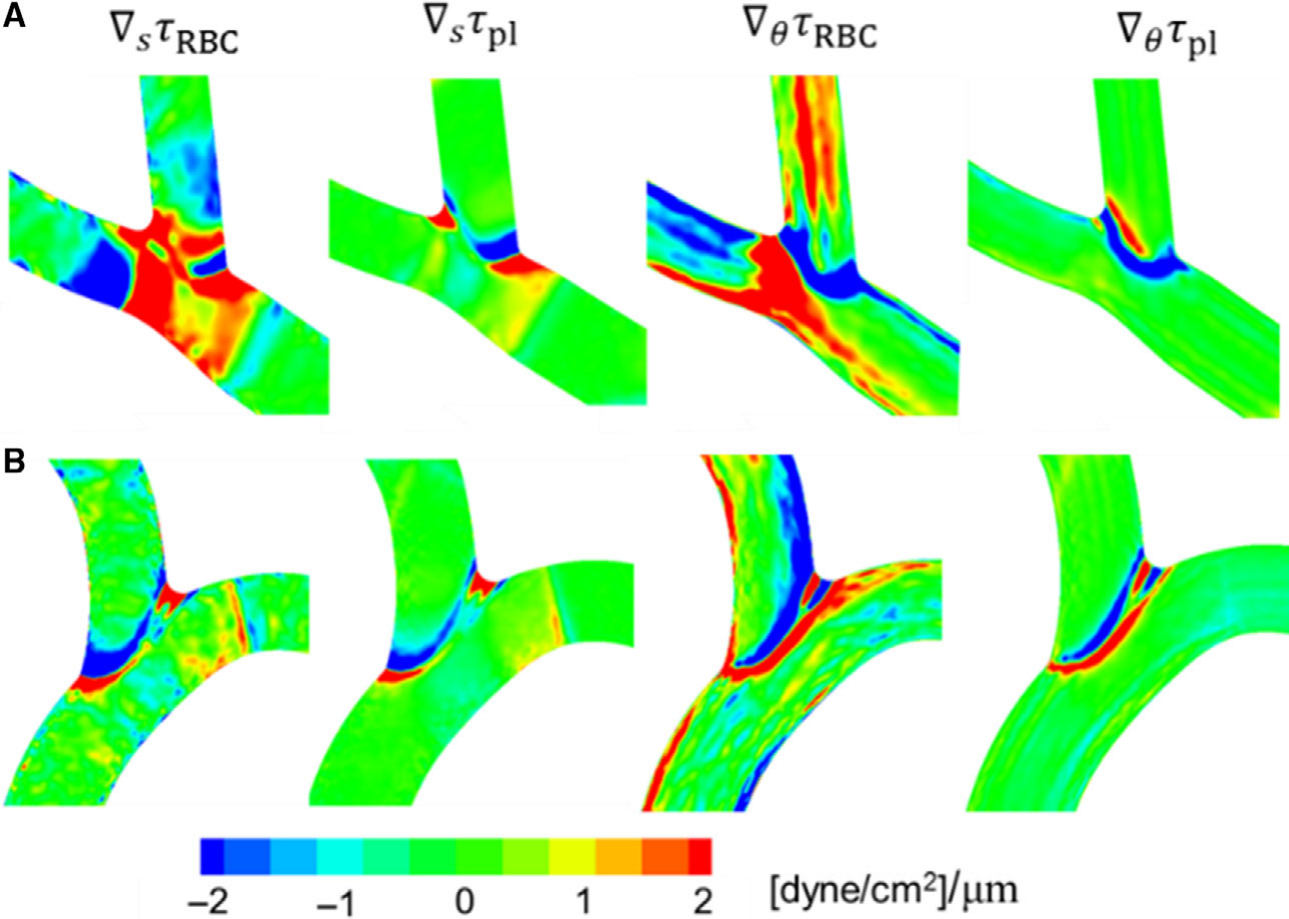
Distribution of WSSG for the bifurcations considered in Figure 15. Shown here are axial and circumferential components of WSSG from simulations with and without RBCs.

WSSG contours for the two example bifurcations are shown in Figure 16. Without the RBCs, larger WSSG magnitudes are observed in narrow regions where the branches emanate. With the RBCs, increased WSSG is observed in wider regions. For smaller bifurcations, the increased WSSG is observed everywhere within the bifurcation region. For larger bifurcations, the increase occurs primarily around the origin of the side branch and at the apex. These patterns also arise following the trends observed for WSS, and due to the way RBCs flow through the respective bifurcations. In both examples in Figure 16, positive and negative gradients exist over short distances.

**Figure 17:**
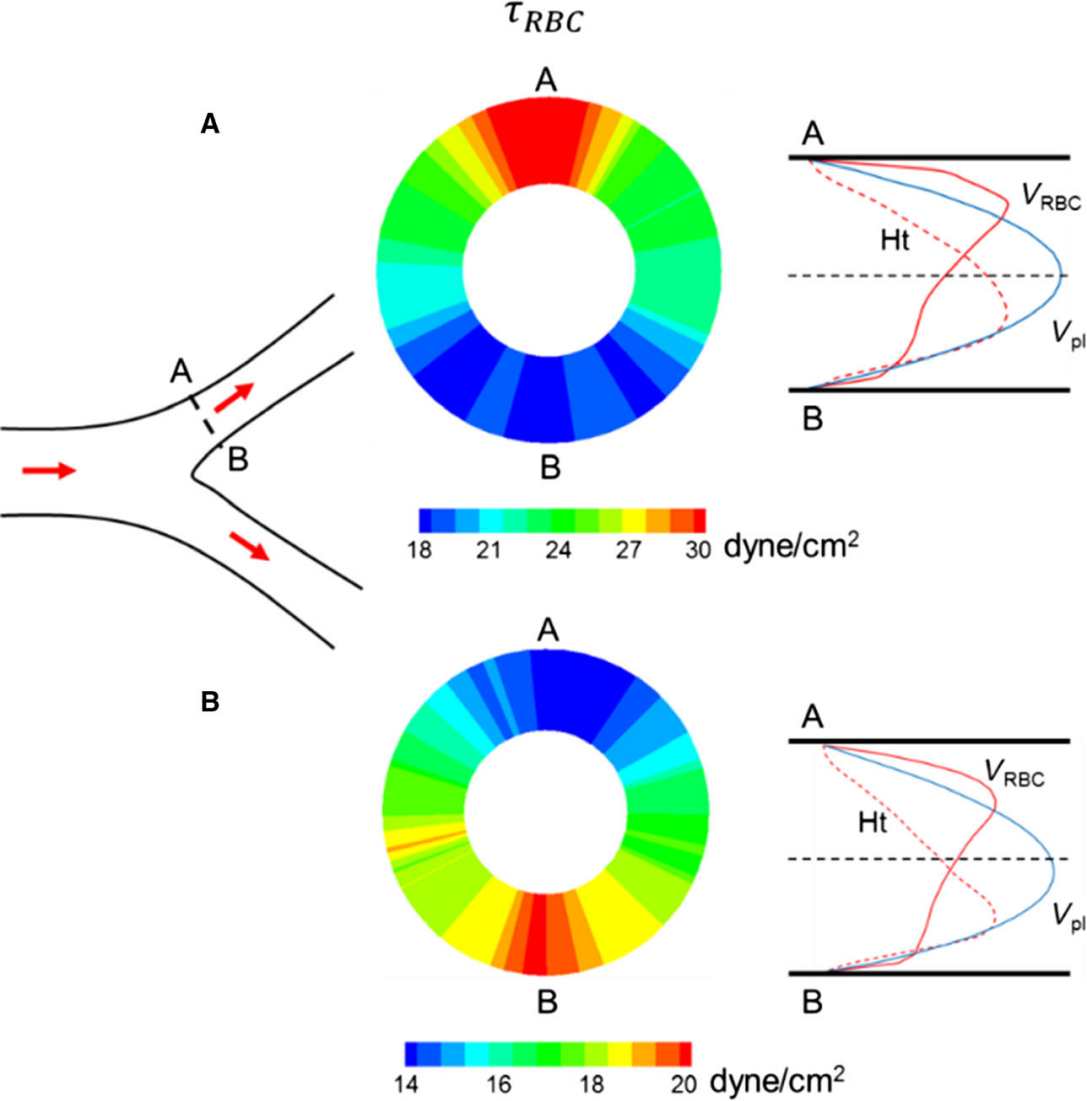
Relationship between velocity profile skewness and WSS skewness. Left: schematic of a bifurcation showing a cross section (A–B) of interest in one daughter vessel. Middle: Cross‐sectional distribution of *τ*_RBC_ in the cross section A–B for two example daughter vessels. Right: Velocity profiles in the presence of RBCs (red curve) and without RBCs (blue curve), and hematocrit profile (dashed red curve). For the vessel shown in (A), *τ*_RBC_ near location A is higher than that near location B, and the velocity profile in the presence of RBCs is skewed toward side A. For the vessel shown in (B), *τ*_RBC_ near location B is higher than that near A, but the velocity profile in the presence of RBCs is skewed toward side A.

An interesting result obtained from our simulations concerns the relationship between the velocity profile skewness and WSS skewness. As blood flows out of a bifurcation, the velocity profile is observed to be skewed toward the side of each daughter vessel that is opposite the bifurcation apex, as shown in Figure 17. This happens because RBCs tend to flow more slowly along the apex‐side of the daughter vessels, as previously noted, thereby reducing the local blood velocity along this side. This is also illustrated in Figure 17 where the plotted hematocrit profiles show a skewed profile toward the side of the vessel nearest the apex. In many small bifurcations, the RBCs severely slow down, and this results in the near‐wall velocity gradients, and hence WSS, being smaller on this apex‐side. As such, for these bifurcations the WSS is highest on the side opposite the apex, which is also the side favored by the velocity skewness (Fig. 17A). In contrast, in larger bifurcations and some smaller bifurcations, the highest WSS occurs on the side that is not favored by the velocity skewness. This latter behavior is illustrated in Figure 17B. This is noteworthy as it is counterintuitive to expect that the highest WSS would occur on the side that is not favored by the velocity profile. This happens when there is less confinement so that RBCs steadily flow, but a thinner cell‐free layer is created on the side closer to the apex resulting in WSS being highest on this side. This interesting relationship is observed due to the presence of the RBCs and their interaction with the geometry. In the absence of RBCs the WSS skewness generally follows the velocity skewness. This can be seen in Figure 17 where for each of the example vessels the velocity profile of pure plasma is skewed toward side B, which is also the side with the higher wall velocity gradient (i.e., higher WSS without RBCs).

#### WSS at venular convergences

**Figure 18:**
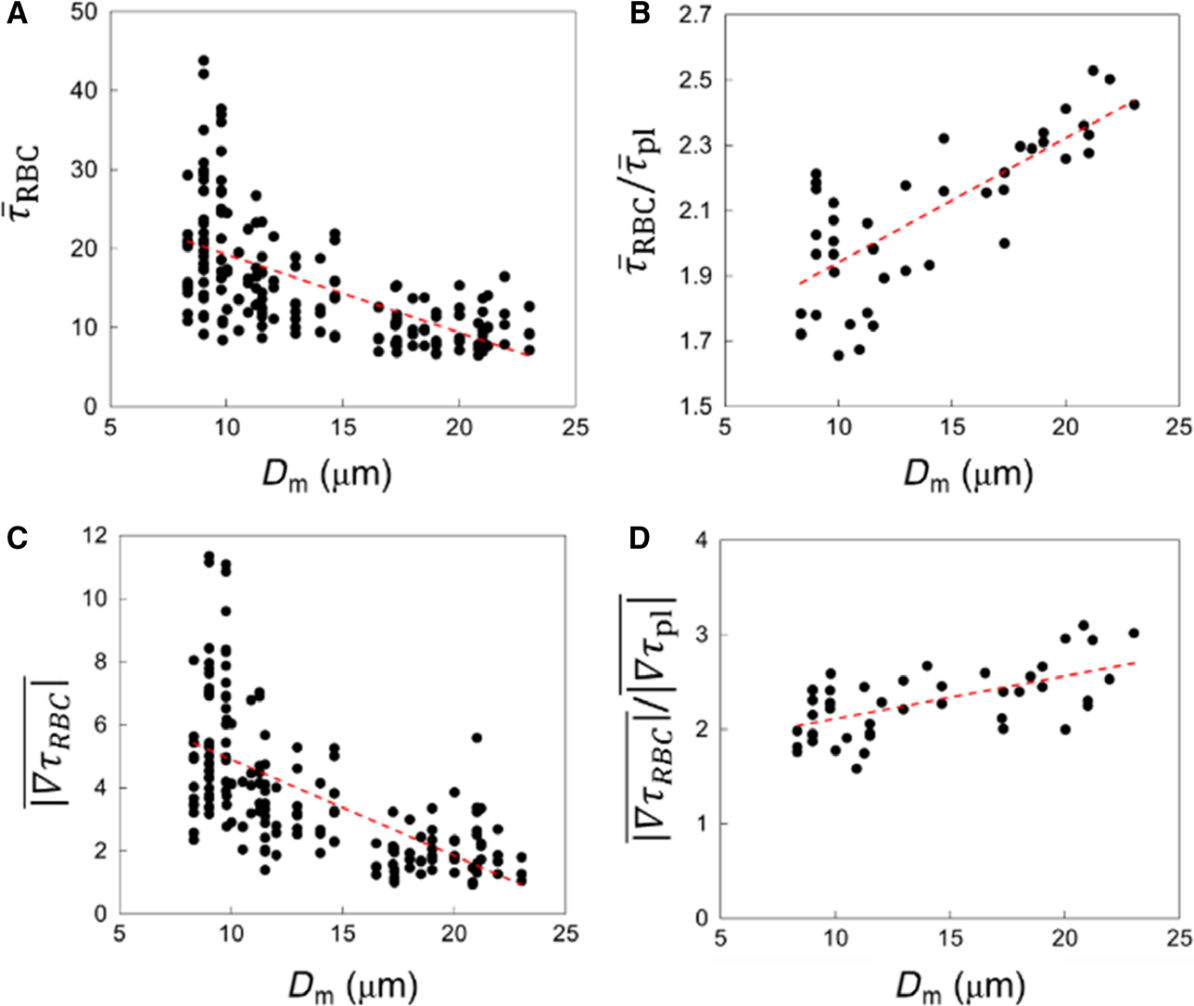
ROI‐averaged data for venular convergence regions. (A) ${\overset{¯}{\tau }}_{\mathrm{RBC}}$, (B) ${\overset{¯}{\tau }}_{\mathrm{RBC}}/{\overset{¯}{\tau }}_{\mathrm{pl}}$, (C) WSSG magnitudes in the presence of RBCs, and (D) ratio of WSSG magnitudes for simulations with and without RBCs, each as functions of collecting vessel diameter(*D*_*m*_). Red lines represent linear regressions.

We now present the results for venular convergences. As shown previously, WSS averaged over all venular convergences is significantly less than that for bifurcations. However, there is a wide distribution among different convergences. This is shown in Figure 18A ${\overset{¯}{\tau }}_{\mathrm{RBC}}$ where is plotted for all convergences as a function of the diameter (*D*_*m*_) of the collecting vessel. Similar to what was observed for bifurcations, ${\overset{¯}{\tau }}_{\mathrm{RBC}}$ for convergences also tends to increase with decreasing *D*_*m*_. The ${\overset{¯}{\tau }}_{\mathrm{RBC}}/{\overset{¯}{\tau }}_{\mathrm{pl}}$ ratio is also larger than one in all convergences, as shown in Figure 18B. However, ${\overset{¯}{\tau }}_{\mathrm{RBC}}/{\overset{¯}{\tau }}_{\mathrm{pl}}$ shows an increasing trend with increasing *D*_*m*_, which is opposite to the trend observed at bifurcations. This happens because ${\overset{¯}{\tau }}_{\mathrm{pl}}$ decreases significantly with increasing *D*_*m*_. WSSG magnitudes are plotted in Figure 18C, and the ratio $\bar{\left|\nabla \tau_{\mathrm{RBC}}\right|}/\bar{\left|\nabla \tau_{\mathrm{pl}}\right|}$ is shown in Figure 18D. $\bar{\left|\nabla \tau_{\mathrm{RBC}}\right|}$ is observed to increase with decreasing *D*_*m*_ due to increasing cellular influence. In contrast, $\bar{\left|\nabla \tau_{\mathrm{RBC}}\right|}/\bar{\left|\nabla \tau_{\mathrm{pl}}\right|}$ shows an increasing trend with increasing *D*_*m*_ since $\bar{\left|\nabla \tau_{\mathrm{pl}}\right|}$ becomes very small, which is also in agreement with the trend observed for bifurcations.

**Figure 19:**
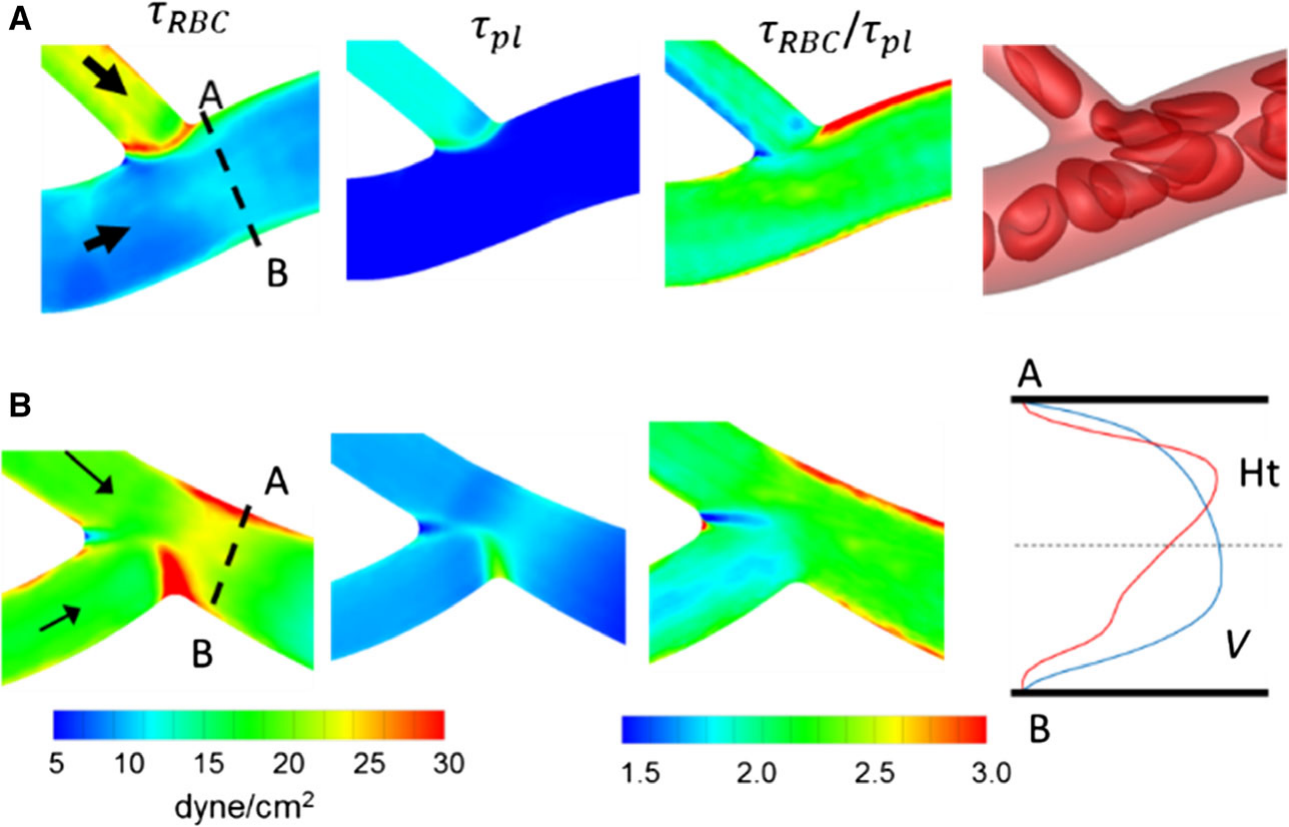
Distribution of WSS at (A) a venular convergence where a capillary vessel (*D* = 6 *μ*m) merges with a larger diameter vessel (*D* = 11 *μ*m), and (B) a convergence where two capillary vessels merge (D = 6 *μ*m). Shown here are WSS with and without RBCs, their ratio, and a snapshot showing RBC flow dynamics (A only). Also shown for (B) are the hematocrit and velocity profiles in the presence of RBCs at the cross‐section A–B.

We now consider the detailed spatial variations of WSS and their underlying mechanisms at representative venular convergences. Figure 19A shows the 3D WSS distribution at a convergence where a smaller capillary vessel merges with a larger diameter venule. *τ*_*RBC*_ is the highest along the edge where the capillary vessel connects with the venule. This is a geometric effect as *τ*_*pl*_ also shows the highest value around this edge. The influence of RBCs is observed at the inlet (marked as A—B) to the collecting vessel where *τ*_*RBC*_ is highest on the sides denoted by A and B. The larger venule feeding the convergence has a steady flow of RBCs in its central core region. As the smaller capillary merges with it, the RBCs from each vessel interact. RBCs from the smaller vessel are forced to side A of the convergence, and those in the larger vessel are displaced toward side B, although to a lesser degree. The overall effect is that WSS is increased on sides A and B, and it is higher on side A than side B. In the absence of RBCs, such spatial variations in WSS are not observed, as is evident from the *τ*_*pl*_ distribution. As such, *τ*_RBC_/*τ*_pl_ shows that the cellular influence is locally enhanced on sides A and B, and more enhanced on side A than B. In contrast, the minimum values of *τ*_*RBC*_ and *τ*_RBC_/*τ*_pl_ occur in the out‐of‐plane regions of the cross section (data not shown)—a trend that follows from the way RBCs flow from the feeding vessels as noted above. While the CFL thickness is reduced along sides A and B as RBCs are pushed to these sides, it is increased on the out‐of‐plane sides. As such, the near‐wall velocity gradient and resulting WSS are the lowest on these sides.

Another example is considered in Figure 19B where a convergence of two capillary vessels is considered. As the diameter of the merging vessels decreases, the enhancement to WSS within convergences increases. *τ*_*RBC*_ is enhanced within the convergence itself because of the increased confinement. This geometric effect is also evident in the *τ*_*pl*_ distribution. The cellular influence is observed at the end of the convergence (marked as A—B), where *τ*_*RBC*_ is higher on side A than B. This occurs because a greater fraction of RBCs flows from the upper feeding vessel than the lower one. Additionally this causes the region of enhanced WSS on side A to be longer than that on side B.

It was noted before that for many bifurcations the side of the outlet vessel that is favored by the velocity profile skewness is actually the side with lower WSS. This is also observed in convergences. Figure 19B provides velocity and hematocrit profiles at the inlet to the collecting vessel for a representative example. The velocity profile is skewed toward side B, but the near‐wall slope is higher at side A resulting in a higher WSS there.

**Figure 20:**
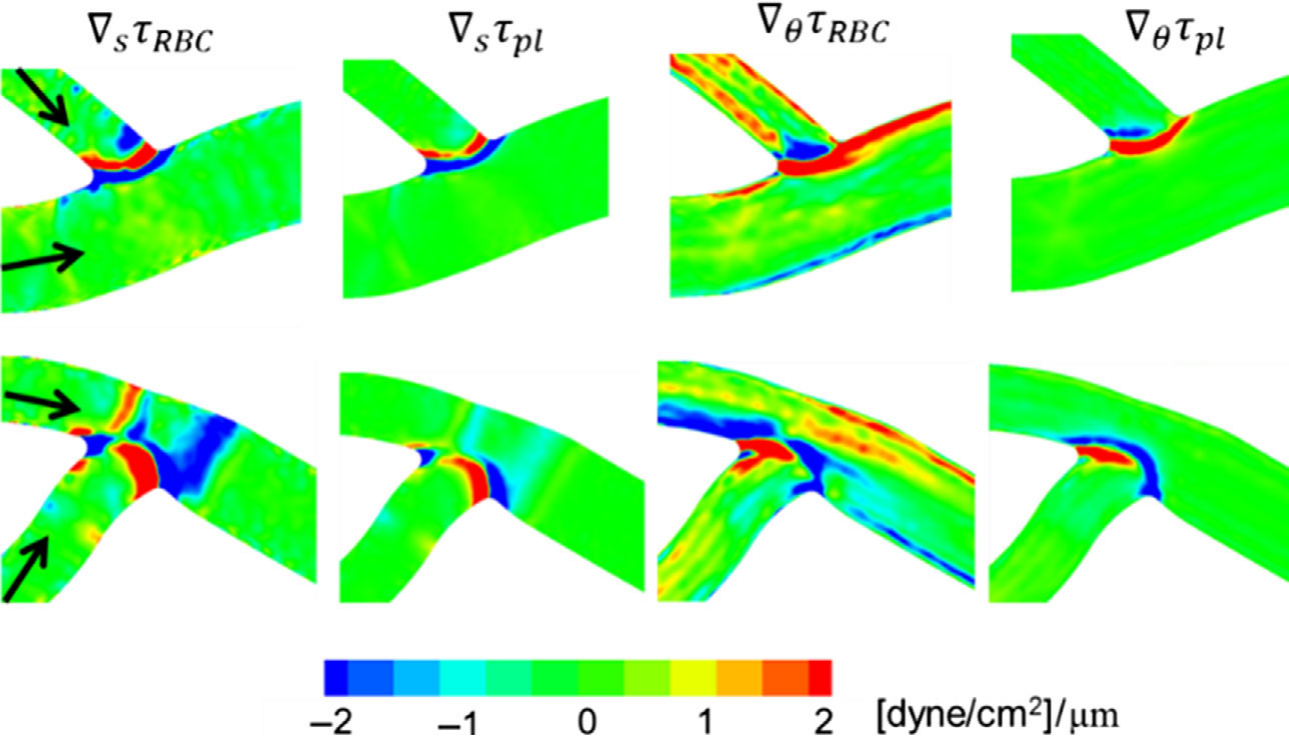
Distribution of WSSG for the venular convergences considered in Figure 19. Shown here are axial and circumferential components of WSSG in simulations with and without RBCs.

Figure 20 shows the WSSG distributions for the two selected convergences. For the plasma‐only simulations, high WSSGs are localized around the edge where the vessels merge. The presence of RBCs causes both axial and circumferential components of WSSG to increase. Furthermore, in the presence of RBCs, high WSSG occurs over wider regions.

## Discussion

Using a 3D computational model of blood flow in microvascular networks, we provide complete 3D quantification of WSS variations and the role of RBCs. In agreement with in vivo studies (Lipowsky et al. 1978; Pries et al. 1990, 1994, 1995a,1995b; Sarelius 1993; Koutsiaris et al. 2007) and recent semiempirical models (Pries et al. 1990, 1995a; Sriram et al. 2014a,2014b) that take into account the non‐Newtonian nature of blood, the present model predicts a significant heterogeneity in WSS from one vessel to another in the networks, in violation of Murray's law. The present model predicts that WSS is significantly higher in precapillary bifurcations and in capillary vessels. Within each group of vascular segments, large ranges of WSS are predicted, and the largest range is observed in capillary vessels where the WSS varies by an order of magnitude. The aforementioned semiempirical models, based on the assumption of 1D flow in vessels, however, do not distinguish between vessels and bifurcations/convergences. This distinction and the resulting differences in WSS in different regions of the vascular networks are important as EC phenotypes differ based on their locations in the vascular tree (Yu et al. 1997; Gerard et al. 1999; dela Paz and D'Amore 2009; Fleming et al. 2017). While EC sensing of WSS in arterioles directly affects flow regulation by SMC activation, capillaries lack SMC. However, vasomotion in capillaries is regulated by pericytes (Hamilton et al. 2010). Recent studies showed that pericytes are sensitive to direct shear stress, and can lead to a modified EC response involved in vascular remodeling (Schrimpf et al. 2015).

The present model provides the complete 3D distribution of WSS throughout each network. Such 3D variations arise due to both network morphology as well as the dynamics of RBCs as they flow through the networks. The 3D patterns of WSS are complex and significant as they can occur on the scale of a few micrometers, which then lead to large values of WSSG. We predict that bifurcation and convergence regions have higher WSSGs with an average magnitude in the range 3–3.5 dyne/cm^2^/*μ*m (3 × 10^4^ dyne/cm^3^). Interestingly, this range of WSSG has also been predicted to occur in the macrocirculation (Dolan et al. 2013), but is significantly higher than that used in vitro to study WSSG response of EC (DePaola et al. 1992; Thi et al. 2004; Ostrowski et al. 2016). For example, Thi et al. (2004) considered WSSG up to 2500 dyne/cm^3^, and observed augmented redistribution of EC cytoskeletal F‐actin. Significantly elevated WSSG predicted by the present model suggests the possibility of significant gradient‐mediated EC activation in localized regions of the vasculature. Studies have shown that EC proliferation increased in regions of high WSSG followed by net migration directed away from these regions, while EC apoptosis increased in regions of low WSSG (Tardy et al. 1997; Ostrowski et al. 2014). ECs in high WSSG regions also experience more significant junctional disruption (Thi et al. 2004). Furthermore, in the macrocirculation WSS variation occurs over the scale of several millimeters (Dolan et al. 2013), whereas the predicted WSS variation in the simulated networks occurs over a few micrometers. Thus, the present model predicts that WSSG in the microcirculation occurs at sub‐EC scale. We predict that both circumferential and axial components of WSSG exist and they are significant. Given that ECs wrap around capillary vessels and extend several tens of micrometers in the longitudinal direction, such sub‐EC scale variations of WSS and WSSG are expected to result in a differential spatial change in membrane fluidity, cytoskeletal organization, and focal adhesion (Wang et al. 1993; Barbee et al. 1995; Tardy et al. 1997; Haidekker et al. 2000; Butler et al. 2001; Barbee 2002; Li et al. 2002; Thi et al. 2004; Chien 2008; Yamamoto and Ando 2013; Fleming et al. 2017), which is also linked to EC mechanotransduction and migration. The present model, however, does not include EC topology along vascular wall, such as the reduction of vessel lumen due to the EC nucleus bulging into the lumen. We expect that such geometric details will cause an even higher magnitudes of WSSG (Barbee et al. 1995; Frame et al. 1998). The present models also do not resolve the glycocalyx. This is an important topic, but a rigorous treatment of the glycocalyx is beyond the scope of the current work. We expect that the spatial patterns of WSS and WSSG, which arise due to the effect of the network geometry and as the “footprints” of RBCs should remain the same with or without the glycocalyx.

Most in vitro studies on EC response to WSS and WSSG considered an RBC‐free medium (DePaola et al. 1992; Barbee et al. 1995; Haidekker et al. 2000; Butler et al. 2001; Li et al. 2002; Thi et al. 2004; Kadohama et al. 2007; LaMack and Friedman 2007; Yamamoto and Ando 2013; Ostrowski et al. 2014). Many theoretical studies on WSS distribution in curved microvessels and bifurcations, and over layers of ECs, did not explicitly include RBCs (Barbee et al. 1995; Noren et al. 2000; Wang and Bassingthwaighte 2003; Liu et al. 2008; Yan et al. 2012). By accurately modeling the deformation and flow dynamics of each individual RBC as they flow through the simulated networks, and by comparing the results against the flow of pure plasma, we show that RBCs strongly influence magnitudes of WSS, WSSG, as well as their distribution patterns. WSS is seen to increase in the presence of RBCs in every region in the networks, while the highest increase by a factor of three is noted in venules. This is intriguing as the WSS itself is the lowest in venules. The origin of enhanced influence of RBCs in venules is shown to be the bluntness of the velocity profile. It may be noted that our model does not include RBC aggregation, which typically occurs in venules due to the low shear. Aggregation was shown to increase the velocity profile bluntness (Bishop et al. 2001). As such, if RBC aggregation is considered in the model, it will have an even greater influence on venular WSS. Within a bending vessel, the influence of RBCs on WSS is higher along the side of the vessel where the WSS itself is lower. Furthermore, the increased WSS at bifurcations and convergences, and its spatial patterns, are shown to be directly related to the flow behavior of RBCs in these regions. It remains to be seen whether such localized variations can be predicted by continuum‐scale models without explicit consideration of individual cells.

We show that RBCs cause a two to four times increase in WSSG. The region of high WSSG is shown to enlarge in the presence of RBCs. In the absence of RBCs, the axial component of WSSG is greater than the circumferential component nearly in every region of the network. In the presence of RBCs, however, the circumferential component is greater than the axial component in most vessels.

While the primary focus of the present work has been on the time‐averaged behavior, we have shown that temporal fluctuations in WSS and WSSG are significant. The RMS fluctuation values for WSS are observed to range anywhere from 2 to 30 dyne/cm^2^, and between 30 and 70% of the mean values for each ROI. More significantly, for WSSG the RMS fluctuation values for the axial and circumferential components are observed to range from 2 to 18 dyne/cm^2^/*μ*m, and can reach upwards of 10 times that of the mean values for vessels. WSSG fluctuations in bifurcations and mergers were less severe than in vessels, with RMS values upwards of six times the mean. In contrast, relative RMS fluctuations in the WSS itself were observed to be higher in bifurcations and mergers than in vessels.

The high absolute RMS values for WSS and WSSG can be explained by the significant temporal variations in hematocrit that arise due to the cell‐cell and cell‐vasculature interactions. These variations are most pronounced in small capillary vessels as RBCs flow in single‐file motion, and intermittently. The timescale of these fluctuations is on the order of milliseconds, and it is possible that this could be physiologically relevant as far as the endothelial cell response is concerned. We also note that while RBC aggregation has not been modeled in the present work, the inclusion of it could potentially increase the magnitude of temporal fluctuations. The recent work of (Brust et al. 2014) suggested the formation of clusters even in capillaries, which could enhance the WSS fluctuations therein.

Our detailed modeling also provides insights on the relationship between velocity profiles and asymmetry in WSS. In general, the velocity profile downstream of a bifurcation or convergence is skewed to one side of the vessel. It is then intuitive to expect that the WSS will be higher on that side as well. On the contrary, our results show that in many precapillary bifurcations and in nearly all convergences, the WSS is higher on the other side that is not favored by velocity profile. This also happens because of the local RBC dynamics in these regions. This finding has implications in experimental determination of WSS where velocity profiles are first constructed from flowing RBCs or other microparticles (e.g., (Kim and Sarelius 2003)). If the vessel diameter is small and RBC or particle centers are not immediately adjacent to the wall, the velocity gradient at the wall, and hence, the WSS, may not be correctly evaluated.

## Conclusions

Using an RBC‐resolved model of blood flow in physiologically realistic microvascular networks, we have presented the full 3D maps of WSS and WSSG, and quantified the influence of RBCs on them. A strong heterogeneity in WSS and WSSG is observed across the networks, with the highest WSS occurring in precapillary bifurcations and capillary vessels. 3D variations of WSS and WSSG are shown to occur on the scale of micrometers due to both network morphology and the influence of RBCs. WSS is seen to increase in the presence of RBCs, with the highest increase being in venules. Within a bending vessel, the influence of RBCs on WSS is higher along the side of the vessel where the WSS itself is lower. WSSG also increases significantly, and high WSSG occurs over wider regions in the networks in the presence of RBCs. RBCs differentially affect the components of WSSG; in most vessels the circumferential component is observed to be greater than the axial component in the presence of RBCs, while the opposite is observed when RBCs are not considered.
